# Women’s Marriage Age Matters for Public Health: A Review of the Broader Health and Social Implications in South Asia

**DOI:** 10.3389/fpubh.2017.00269

**Published:** 2017-10-18

**Authors:** Akanksha A. Marphatia, Gabriel S. Ambale, Alice M. Reid

**Affiliations:** ^1^Department of Geography, University of Cambridge, Cambridge, United Kingdom

**Keywords:** women, marriage age, public health, demography, education, geography, South Asia

## Abstract

In many traditional societies, women’s age at marriage acts simultaneously as a gateway to new family roles and the likelihood of producing offspring. However, inadequate attention has previously been given to the broader health and social implications of variability in women’s marriage age for public health. Biomedical scientists have primarily been concerned with whether the onset of reproduction occurs before the woman is adequately able to nurture her offspring and maintain her own health. Social scientists have argued that early marriage prevents women from attaining their rightful education, accessing employment and training opportunities, developing social relationships with peers, and participating in civic life. The aim of this review article is to provide comprehensive research evidence on why women’s marriage age, independent of age at first childbirth, is a crucial issue for public health. It focuses on data from four South Asian countries, Bangladesh, India, Nepal, and Pakistan, in which marriage is near universal and where a large proportion of women still marry below the United Nations prescribed minimum marriage age of 18 years. Using an integrative perspective, we provide a comprehensive synthesis of the physiological, bio-demographic, and socio-environmental drivers of variable marriage age. We describe the adverse health consequences to mothers and to their offspring of an early age at marriage and of childbearing, which include malnutrition and high rates of morbidity and mortality. We also highlight the complex association of marriage age, educational attainment, and low societal status of women, all of which generate major public health impact. Studies consistently find a public health dividend of increased girls’ education for maternal and child nutritional status and health outcomes. Paradoxically, recent relative increases in girls’ educational attainment across South Asia have had limited success in delaying marriage age. This evidence suggests that in order for public health initiatives to maximize the health of women and their offspring, they must first address the factors that shape the age at which women marry.

## Introduction

United Nations (UN) Conventions and Resolutions consider “child, early, and forced marriage” as a fundamental violation of human rights (1). Marriage before 18 years is considered to be a harmful practice because it denies girls the right to the highest attainable standard of general, sexual, and reproductive health, and to a life free from violence (1, 2). Under-age marriage also constrains evolving physical, emotional, and personal maturity required to safely and successfully transition to adulthood (3, 4). It places restrictions on opportunities in life, such as the right to education. Under-age marriage also restricts women’s ability to fully participate in family, socio-cultural, and civic activities (3). Collectively, these consequences have major implications for public health.

Several UN agreements define parameters relating to marriage and reproduction, including establishing a minimum allowable marriage age. Women have equal rights to men to “freely chose a spouse and to enter into marriage only with their free and full consent” (1). Women also have the right to good reproductive and sexual health. This includes a satisfying and safe consensual sexual experience, the capability to reproduce, and the freedom to decide if, and when, to bear a child. Access to timely and adequate health care for women and their children is also essential (5). Since marriage entails adult responsibilities and also understanding of its consequences, setting a minimum age is a legal guarantee that adult responsibilities are not assigned to children prematurely (6). The age at which legal majority or adulthood is reached is thus important for establishing a minimum age of marriage: Human Rights Conventions set both at 18 years (3, 7).

By ratifying these international agreements, governments are expected to legislate a minimum age at marriage for both sexes, ideally at 18 years. Globally, however, it ranges from 10 to 20 years, meaning legal protection is often not offered to children when the majority status of “adulthood” is reached *via* marriage before 18 years (6). In absolute terms, only 11 countries have established a minimum legal marriage age at 18 years without any dispensation; 73 have an ascribed minimum age but allow exceptions below 18 years, usually for girls; and 102 have unclear information or no established minimum marriage age (6).

### Scale and Geographic Distribution of Women’s Under-Age Marriage

In many low- and middle-income countries, a greater proportion of females than males marry “under-age,” or below the UN legal threshold of 18 years. In 2011, an estimated 720 million women aged 18 years or older were married under-age compared with 156 million boys (8). The reasons for which the two sexes marry under-age most likely differ and merit appropriate consideration. However, in this review of public health implications, we focus on why girls marry under-age.

Between 2000 and 2011, one in three women aged 20–24 years in the global south (excluding China) were estimated to have married before they reached the age of 18 years (9). In 2010, this was equivalent to nearly 67 million women, with approximately one in nine or 12% marrying as children, before the age of 15 years (9). At the current rate, 39,000 girls are projected to marry under-age age each day, amounting to over 14.2 million girls each year over the next decade (9).

**Figure 1:**
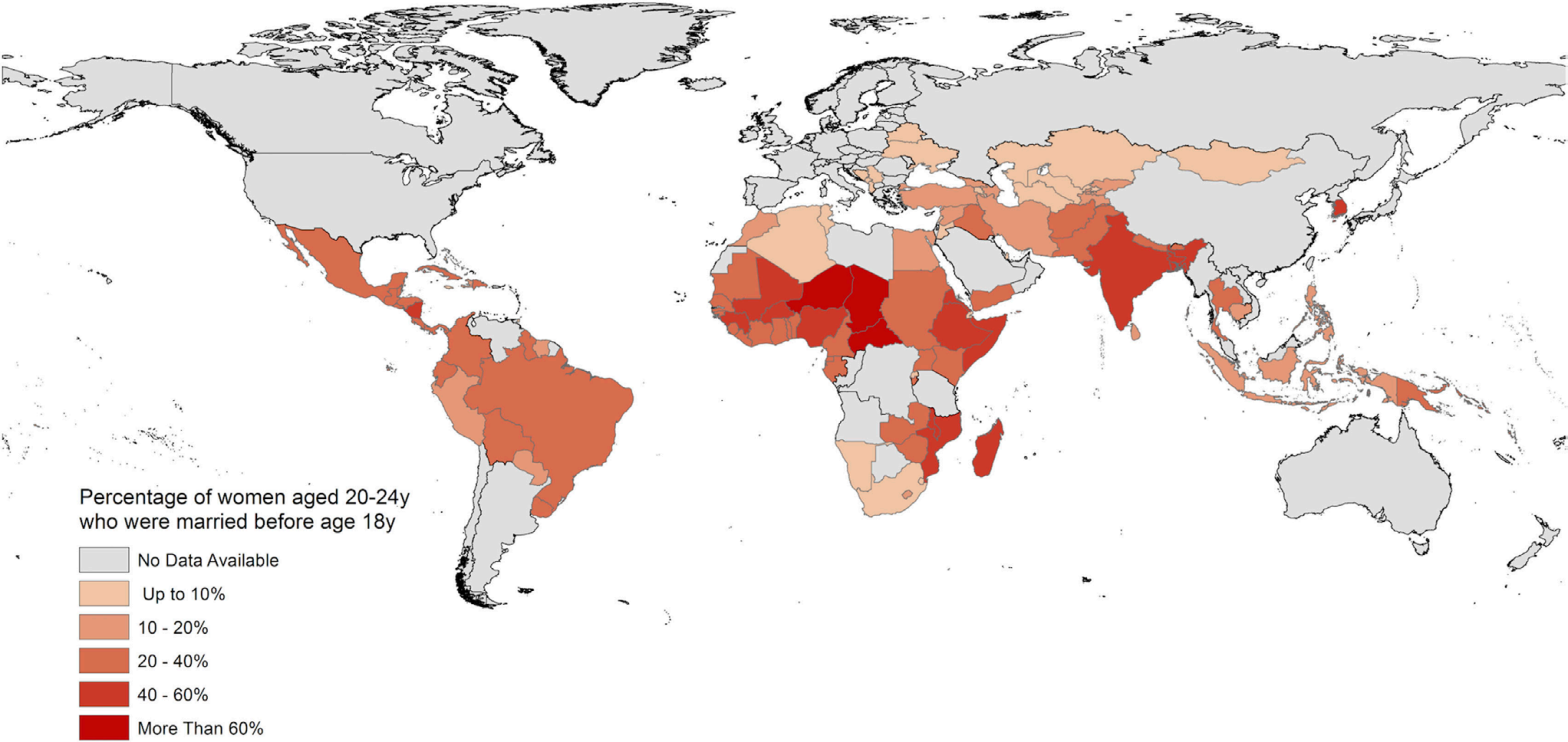
Global distribution of women aged 20–24 years married below the United Nations prescribed minimum age of 18 years. Data compiled from Demographic Health Surveys reports in 2016 by Ref. (10).

Figure 1 illustrates the global geographical distribution of women aged 20–24 years married “under-age.” The data used to produce this map was compiled by the United Nations Children’s Fund in May 2016 from national Demographic Health Surveys (DHS), Multiple-Indicator Cluster Surveys, and other nationally representative surveys conducted between 2008 and 2014 (10). The region with the highest national prevalences comprises central Africa, however, in absolute terms nearly half of all under-age marriages worldwide occur in South Asia.

Weak political recognition of “under-age” marriage and its corresponding high prevalence for girls is a crucial issue for public health. While its importance is recognized across different academic fields, they approach the issue from contrasting perspectives. For example, social scientific research identifies how early marriage is associated with adverse human capital outcomes such as limited opportunities for personal and educational development. Yet the failure of education, the key intervention used to delay girls’ marriage age, is stark. Although 60% of girls in South Asia now attend secondary school, over half still marry before 18 years (11).

**Figure 2:**
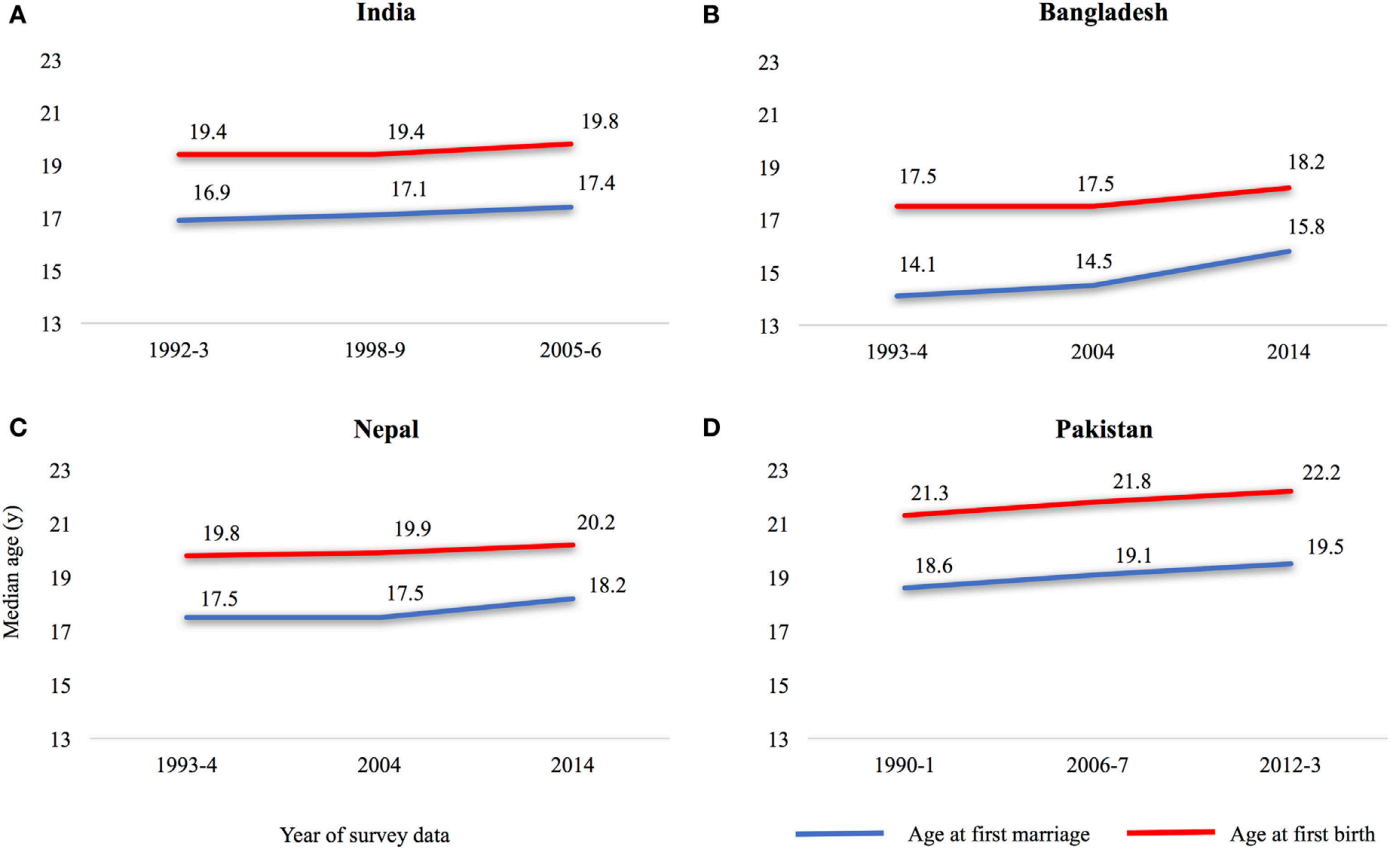
Median age at first marriage and at childbirth, women aged 25–49 years in South Asia, **(A)** India, **(B)** Bangladesh, **(C)** Nepal, and **(D)** Pakistan. Adapted and redrawn with permission from Ref. [(16), Figure 1A].

In contrast, demographic and public health research focus on *early age at childbirth* as the key event in women’s lives leading to multiple adverse maternal and child health outcomes. However, in traditional societies childbirth usually *follows soon* after marriage (12, 13). Figures 2A–D are adapted and redrawn from DHS data produced by MacQuarrie on women aged 25–49^1^ years. In the four South Asian countries with the highest prevalence of under-age marriage, first childbirth occurred on average 2.5 years after marriage (16). Marriage age remains the most consistent influence on the first birth interval, even after controlling for birth cohort, gendered context^2^, spousal educational attainment, and socio-economic characteristics (16). This evidence suggests that the key decision which needs to be delayed in this population is marriage age, which will invariably lead to an older age at childbirth.

### Aims and Methodology

The aim of this review is to provide comprehensive research evidence on why women’s marriage age, independent of age at first childbirth, is a crucial issue for public health. We go beyond what has previously been done by synthesizing key insights and inter-linkages from the demographic, health, and human capital literatures. Our novel contribution is to demonstrate that women’s under-age marriage is the “gateway” to the detrimental trans-generational consequences of early childbearing. Marriage age is also a marker of women’s human capital and overall status in society. We seek to share new knowledge on why four South Asian countries, Bangladesh, India, Nepal, and Pakistan, have the highest global prevalence of girls’ under-age marriage.

We searched relevant databases (e.g., PubMed and Eric) for empirical research and review articles in international peer-reviewed journals. We also conducted a broader search (using Google scholar and Google) for gray policy literature published by international development organizations, the UN and national governments on research, legislation, and secular trend data on women’s marriage age. Given the cross-disciplinary approach of our review, we focused on papers that were relevant to public health, demography, and also the social predictors and consequences of women’s marriage age. We searched for papers using the following key terms relating to girls/women: “child marriage,” “early marriage,” “adolescent marriage,” “under-age marriage,” and their inter-linkage with “adolescent pregnancy and health,” “women’s reproductive/sexual, mental health,” “violence against women,” “fertility,” “maternal and child nutritional status/mortality,” “education,” “socio-cultural norms,” “poverty/dowry/economic status,” “women’s social status,” “autonomy,” and “empowerment.” Given the breadth of literature available, our review does at times draw on studies beyond South Asia. Some of our findings are likely to apply more widely. Others might relate to the socio-cultural context of marriage.

A complexity in understanding these inter-linkages is the inconsistent disaggregation of age-categorizations across studies. We address this by adopting a dual spatio-temporal approach. Data on the previous generation of women aged 25–49 years enables us to illustrate secular changes in marriage age and childbearing, and also to emphasize the consequences and benefits conferred to variable marriage age. Data on the most recent cohort of women aged 20–24 years who married below 18 years provides critical insights on the penalties of marrying young in contemporary societies.

There are four sections to this review. Section “Marriage in South Asia” sets out the social context of marriage. It also describes changes in the prevalence of under-age marriage in the four South Asian countries of our review. Section “Consequences of Women’s Under-Age Marriage” provides an integrated perspective on the broad demographic, health, and human capital consequences of early marriage. Section “Predictors of Under-Age Marriage” provides new insights from this diverse literature on the drivers of variability in marriage age. We recognize that separating the consequences from the predictors is in part artificial because of the potential two-way direction of association. However, this approach enables us to critically assess why the high prevalence of under-age marriage persists despite increasing knowledge of its consequences. In Section “Discussion,” we discuss the opportunities and challenges flowing from these mutual fields of interest for research and practice.

## Marriage in South Asia

In the geographical region of South Asia, complex cultural and religious dynamics set parameters around marriage. Generally, for both sexes, marriage is perceived as an essential stage in the life-course and there are strong social sanctions for childbearing outside of marriage (17). As a social institution, marriage is identified by some studies as near universal (18). Generally, any variation relates to the age at which marriage takes place, rather than whether it happens at all. Women also tend to marry younger than men. For example, about 90% of women aged 15–49 years were married by ages 25–29 years in Bangladesh, India, and Nepal compared with 80% of men; marriage is nearly universal among women aged 30 and above and men aged 45 and above (14, 15, 19, 20).

**Figure 3:**
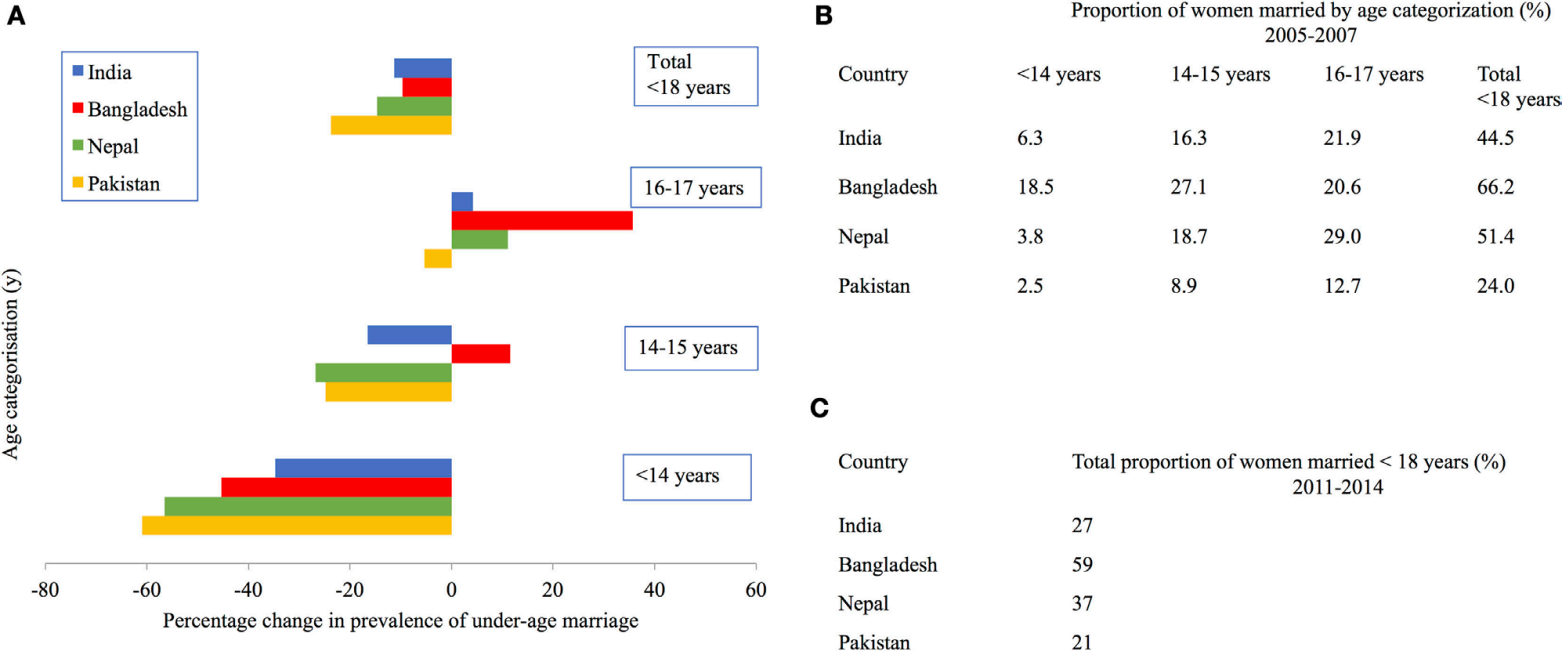
Prevalence of under-age marriage among women aged 20–24 years in South Asian countries. **(A)** Change in prevalence between 1991 and 2007, **(B,C)** prevalence in 2005–2007 and 2011–2014, respectively. Data for **(A,B)** were taken from Ref. [(22), Table 2], for **(C)** from Ref. (14, 15, 20, 21).

Recent DHS data from 2011 to 2014 show that Bangladesh, India, Nepal, and Pakistan have the highest prevalence of women aged 20–24 years marrying under-age (59, 27, 37, and 21, respectively) (14, 15, 20, 21). This translates into tens of millions of girls in each of these countries. Further distinctions in the age at marriage are important to recognize because changes in the prevalence of “child” (<14 years of age) marriages and those taking place during “early-adolescence” (14–15 years) and “late-adolescence” (16–17 years) have differed over the past two decades in these countries. Figure 3A uses DHS data produced by Raj et al. to show that between 1991 and 2007, the overall prevalence of marriage below 18 years in women aged 20–24 years decreased. This was largely attributed to fewer marriages below 15 years. The change in the prevalence of marriages at 16–17 years varied across the four countries. There was a marginal decrease in Pakistan and a slight increase in India. However, the proportion of girls marrying in late-adolescence increased substantially in Nepal and Bangladesh (22). Figure 3B also uses Raj’s et al.’s data from 2005 to 2007 to show that the net effect is that under-age marriage is concentrating in a slightly older age range, but still below 18 years. These patterns are important to recognize because the predictors and consequences of marriage in these different age groups are likely to be different. Figure 3C uses the most recent DHS data from 2011 to 2014 to show the total prevalence of girls marrying <18 years is reported to have decreased further in these countries (14, 15, 20, 21). Disaggregated data by age groups were not yet available. While this trend is promising, a large proportion of women still marry soon *after* 18 years. These women may experience some of the consequences of those who married under-age, in late-adolescence.

Despite having ratified international conventions protecting the rights of children and women generally, many South Asian countries have not ratified agreements directly addressing under-age marriage and the universally ascribed minimum age of 18 years. Table 1 uses data produced by the UN Office for the High Commissioner and the international advocacy group, *Girls Not Brides*, on the ratification status of these international agreements (23–25). Differences between international and national laws suggest that women’s marriage age is, above all, culturally defined.

**Table 1:** International agreements and national law on minimum marriage age and marriage and birth registration in South Asia.

	Bangladesh	India	Nepal	Pakistan
**Key international Conventions and Resolutions relating to marriage or child protection and ratification date^a,b^**				
Convention on the Consent to Marriage, Minimum Age for Marriage and Registration of Marriages (1962)^c^	1998	Not ratified	Not ratified	Not ratified
International Covenant on Economic, Social, and Cultural Rights (1966)^d^	1998	1979	1991	2008
Convention on the Elimination of All Forms of Discrimination Against Women (1979)^d^	1984	1993	1991	1996
Convention on the Rights of the Child (1989)^d^	1990	1992	1990	1990
Resolution on Child, Early, and Forced Marriage (2014)^e^	Not adopted	Not adopted	Not adopted	Not adopted
**National legislation on minimum marriageable age**				
National legislation^f^	Child Marriage Restraint Act (1929); Child Marriage Prevention Act (2014)	Prohibition of Child Marriage Act (2006)	Marriage Registration Act 2028 (1971)	Child Marriage Restraint Act (1929); Muslim Family Law Ordinance (1961)
Statutory law on marriage and majority age^f^	In 2017, “special circumstances” for girls marrying at any age, previously 18 years girls 21 years boys. Majority age both sexes 18 years	Marriage age 18 years girls 21 years boys. Majority age both sexes 18 years	Marriage age 20 years both sexes. Majority age both sexes 18 years	Marriage age 16 years girls 18 years boys. Majority age both sexes 18 years
Customary/religious law on marriage age^f^	Special Marriage Act (1872) non-religious marriage, parental consent; Muslim Personal Law 14 years girls	Sharia and Mohammedan Law with parental consent 15 years girls	National Code with parental consent both sexes 18 years	Child Marriage Act (1872) non-religious union parental consent 14 years girls
**Registration of marriage and births of children under 5 years of age (years)**				
Requirements for registering marriage^f^	Optional, Hindu Marriage Act; Required, Christian, Muslim laws	Mandatory	Mandatory	Mandatory
Percentage of births registered	19% rural, 23% urban (2014)^g^	76% rural, 89% urban (2015–2016)^h^	42% rural, 44% urban (2011)^i^	23% rural, 59% urban (2012–2013)^j^

*^a^A convention is a treaty or formal agreement between States, which, if ratified, is legally binding; States must adhere to creating legal rights and duties according to this instrument (167). ^b^A resolution is a formal expression of views of Member States which is not legally binding; the implementation of policy recommendations is the responsibility of each Member (28). Data from Ref. ^c^(25), ^d^(23), ^e^(24), ^f^(26), ^g^(20), ^h^(21), ^i^(15), and ^j^(14)*.

Table 1 shows that national secular legislation allows marriage at 16 years in Pakistan, 18 years in India, and 20 years in Nepal (26). However, Sharia and Mohammedan law permit marriage for girls at 14–15 years in Bangladesh, India, and Pakistan (26). In Bangladesh, an international debate ensues over a new Act approved by Parliament in 2017 to allow marriage below 18 years in “special cases,” ostensibly omitting obligation to a minimum allowable marriage age for girls (29). Weak national marriage and birth registration systems mean that even the current high prevalence of under-age marriage and rates of adolescent fertility are likely to be under-estimated (14, 15, 20, 21, 26).

## Consequences of Women’s Under-Age Marriage

### Demographic Consequences

This section focuses on the association between under-age marriage and demographic outcomes of fertility and population growth and its related implications for sex-selective abortion and contraception. Implications for maternal and child mortality are addressed in the following section on health consequences. The mechanisms through which these effects operate relate partly to exposure and opportunities for getting pregnant, partly through generation length, and partly through biological, behavioral, and socio-economic factors. Simulations from ecological analyses of 97 countries suggest that a 10% increase in girl child marriage would be associated with a 3% increase in the infant mortality rate, a 0.3% increase in the total fertility rate, a 70% increase in the maternal mortality ratio, and a 10% decrease in skilled birth attendance (30). The magnitude of this problem is large in the South Asia region, as demonstrated below.

In South Asia, unlike many other parts of the world, marriage is still the main context for sexual intercourse. Getting married therefore signals the start of exposure to the chance of becoming pregnant and the earlier a woman gets married, the longer she will spend exposed during her fertile years. Studies find that in the absence of modern contraception, the age at which women marry is the main determinant of the number of children each woman will have (31). Marriage age also plays a very important role in lowering fertility levels from the biological maximum. The availability of reliable contraception offers the chance to thwart this relationship by stopping at a particular desired number of children or by increasing the spacing between births. However, simulations have shown that women who marry young still have more children at the end of their reproductive careers because there is more time for them to increase their desired numbers of births and more opportunity for contraceptive failure to increase fertility (32).

#### Fertility

**Figure 4:**
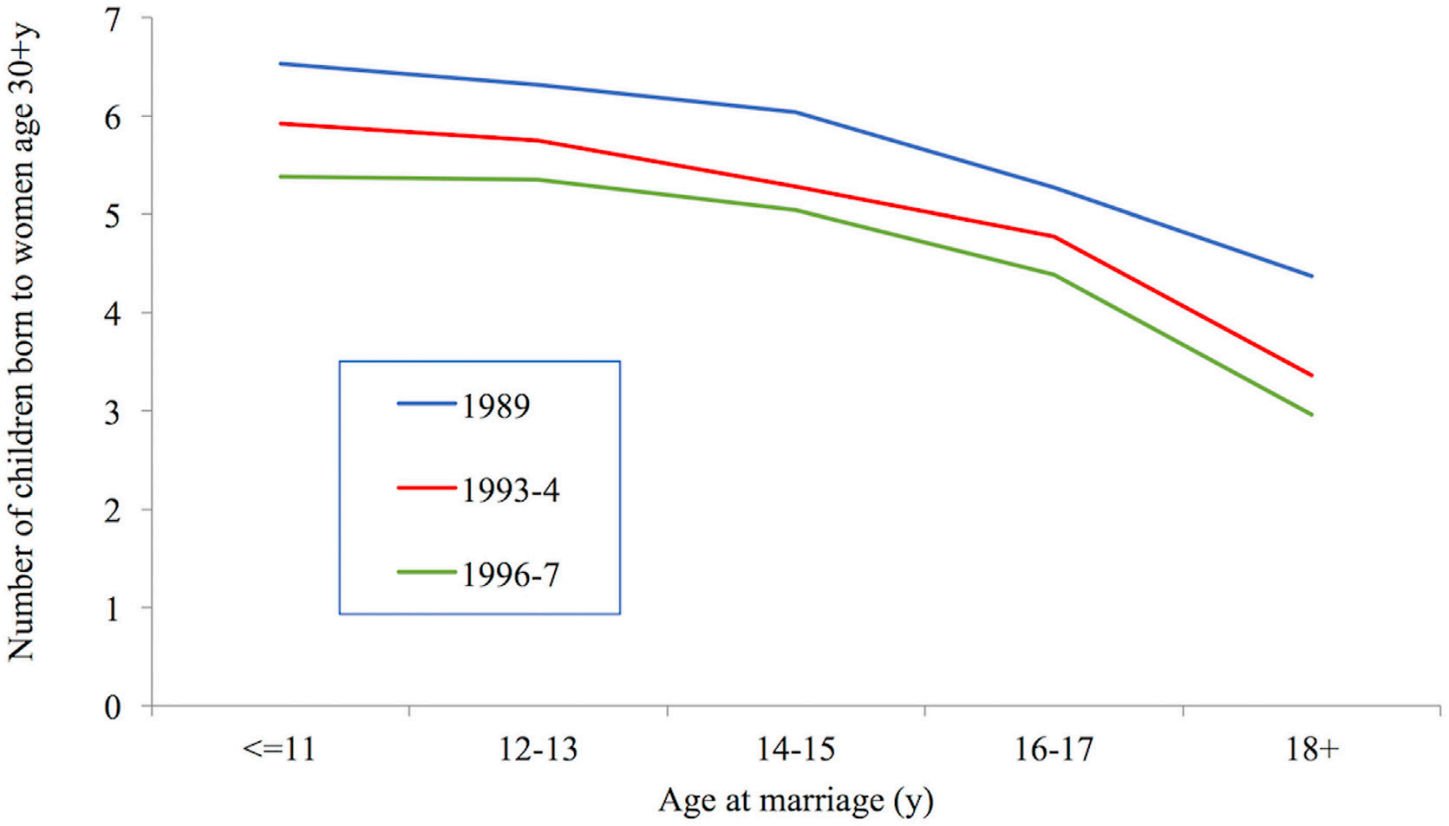
Number of children born to women age 30+ years, Bangladesh 1989–1997. Data taken from Ref. (34).

Empirical studies find that despite some teenage sub-fecundity, early marriage is associated with higher completed fertility at the end of the childbearing years.^3^ Figure 4 shows Bangladesh Fertility Survey and DHS data produced by Kabir et al. which demonstrate that in each of three successive surveys in late 20th century Bangladesh, the younger a woman had married the more children she had produced by the age of 30 years (34). The reference value in the figure is women aged 20–34 years. Nahar et al.’s study confirms the persistence of this trend: among women aged 50 years in the 2007 DHS survey of Bangladesh, those who had married at 19 years or over had on average 2.62 children compared with 3.55 among those who had married between 17 and 18 years, and 4.59, 5.53, and 6.36 among those who had married at 15–16, 13–14, and 12 years and under, respectively (35). Similarly, Adhikari demonstrated that among women aged 40–49 years in the 2006 Nepal DHS, those who had married at 16 years or older had on average 4.7 children compared with 5.3 among those who married before 16 years (27).

So far, we have discussed the association of age at marriage on the number of children born to each woman. However, age at marriage can have a strong effect on a country’s fertility rate even if there are no differences in completed fertility by age at marriage. This is because younger marriage means younger childbearing, and younger childbearing means reduced generation length and more women able to have children at any one time. This increases the crude birth rate which has a positive impact on population growth. Coale and Tye calculated the impact of shifting the age patterns of childbearing from those existing in India in 1956, where fertility was highest in the 20–24 year old age group, to those experienced by the Singapore Chinese population, where fertility was highest in the 25–29 year old age group. Over the course of 10 years this would lower the crude birth rate by 8% without any change in the mean number of children born per woman, simply by increasing the mean generation length by 2.7 years (36).

#### Childbearing and Access to Contraception

**Figure 5:**
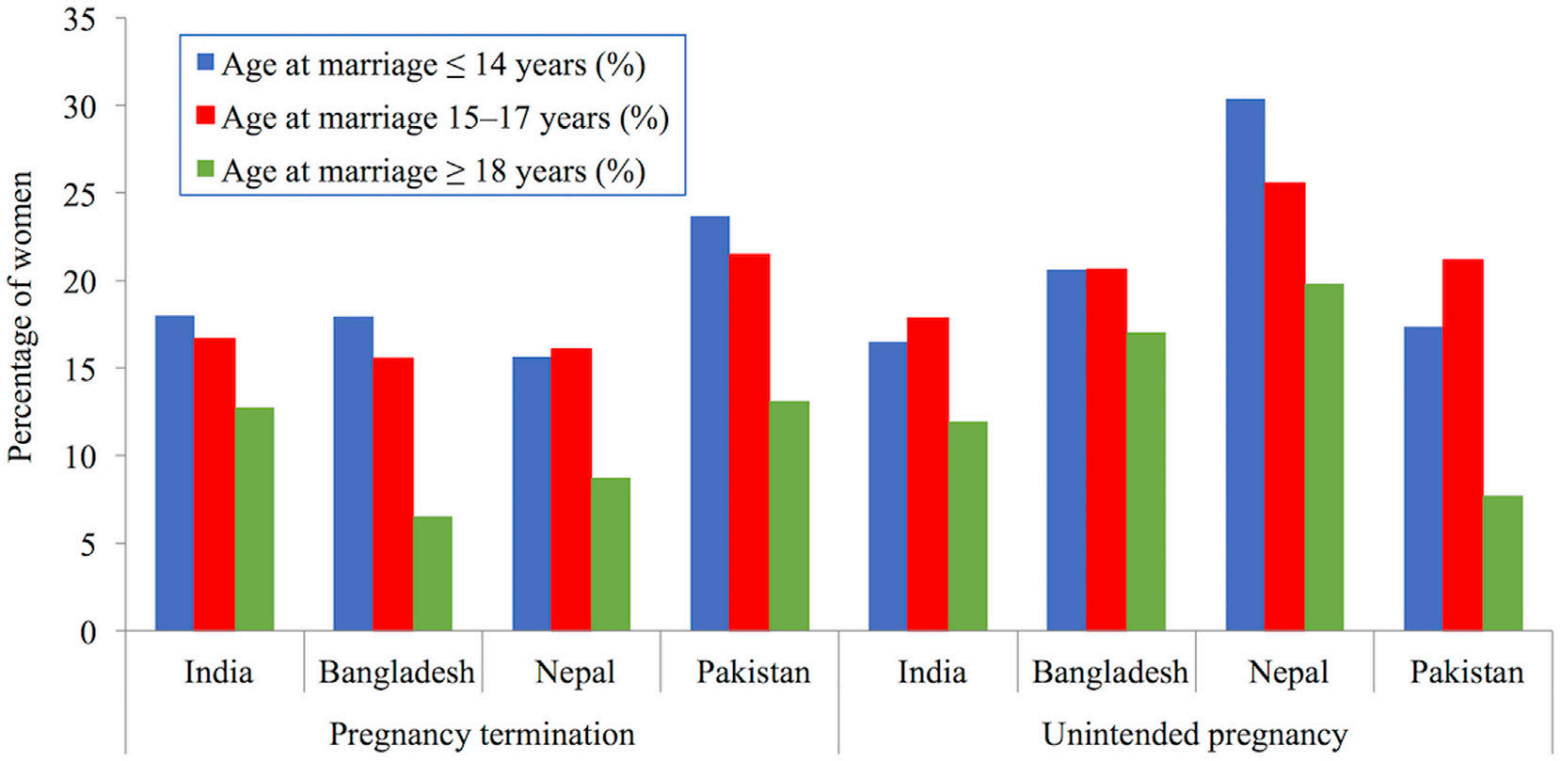
Percentage of women aged 20–24 years experiencing indicators of poor birth control (pregnancy termination and unintended pregnancy), by age of marriage in South Asia. Demographic Health Surveys data used from following surveys: India (2005–2006), Bangladesh (2007), Nepal (2006), and Pakistan (2006–2007). Data taken from Ref. [(39), Table 2].

Comparisons of completed fertility are informative, but because they relate to women who married several decades ago, they may already represent a picture that is out of date. Other studies therefore compare the speed of childbearing among younger women. Raj et al.’s study of 20–24 year old women in India (37) and Nasrullah et al.’s of 20–24 year old women in Pakistan (38) demonstrated shorter birth intervals (i.e., more rapid childbearing) among women who had married before age 18 years than among later marriers, although this is not universally found. Godha et al.’s comparative study of the four South Asian countries considered here did not support a higher pace of childbearing among early marriers (39). A faster pace of childbearing could be the consequence of higher desired fertility among women who marry young, or among their husbands. Additional plausible explanations for faster childbearing include poorer contraceptive knowledge, access to other birth control methods and less control over family planning decisions. These explanations are supported by Figure 5 which uses DHS data produced by Godha et al. to show that women who married early had more unplanned pregnancies and more terminations of pregnancy, which are firmer indicators of poor contraceptive knowledge, access, and control (39). The survey data come from the following countries and years: India (2005–2006), Bangladesh (2007), Nepal (2006), and Pakistan (2006–2007). Nasrullah et al.’s study revealed that these differences remained even after controlling for husbands’ fertility desires and son preference (38). Similar results were obtained in a further study of Bangladesh, although this study is not strictly comparable as it used a wider age range of women (40).

Women in India and Pakistan who marry early are therefore likely to have poorer access to and control over contraception, to have children quicker, and to have more unplanned (or unwanted) children. Child brides from India are also four times more likely than later marriers to have been sterilized by the age of 20–24 years (37). The higher likelihood of termination among early marriers may also be linked to strong son preference and sex selection (37, 38, 40, 41). While these are effective ways of preventing further unplanned pregnancies, they have also been linked to lower female autonomy. Reduced condom use also puts women’s sexual health at risk by increasing the chance of contracting sexually transmitted infections (42).

The following section relates to the health implications of some of these demographic outcomes for maternal and child health, nutritional status, and survival.

### Maternal Outcomes

#### Access to Healthcare and Pregnancy- and Childbirth-Related Morbidity

**Figure 6:**
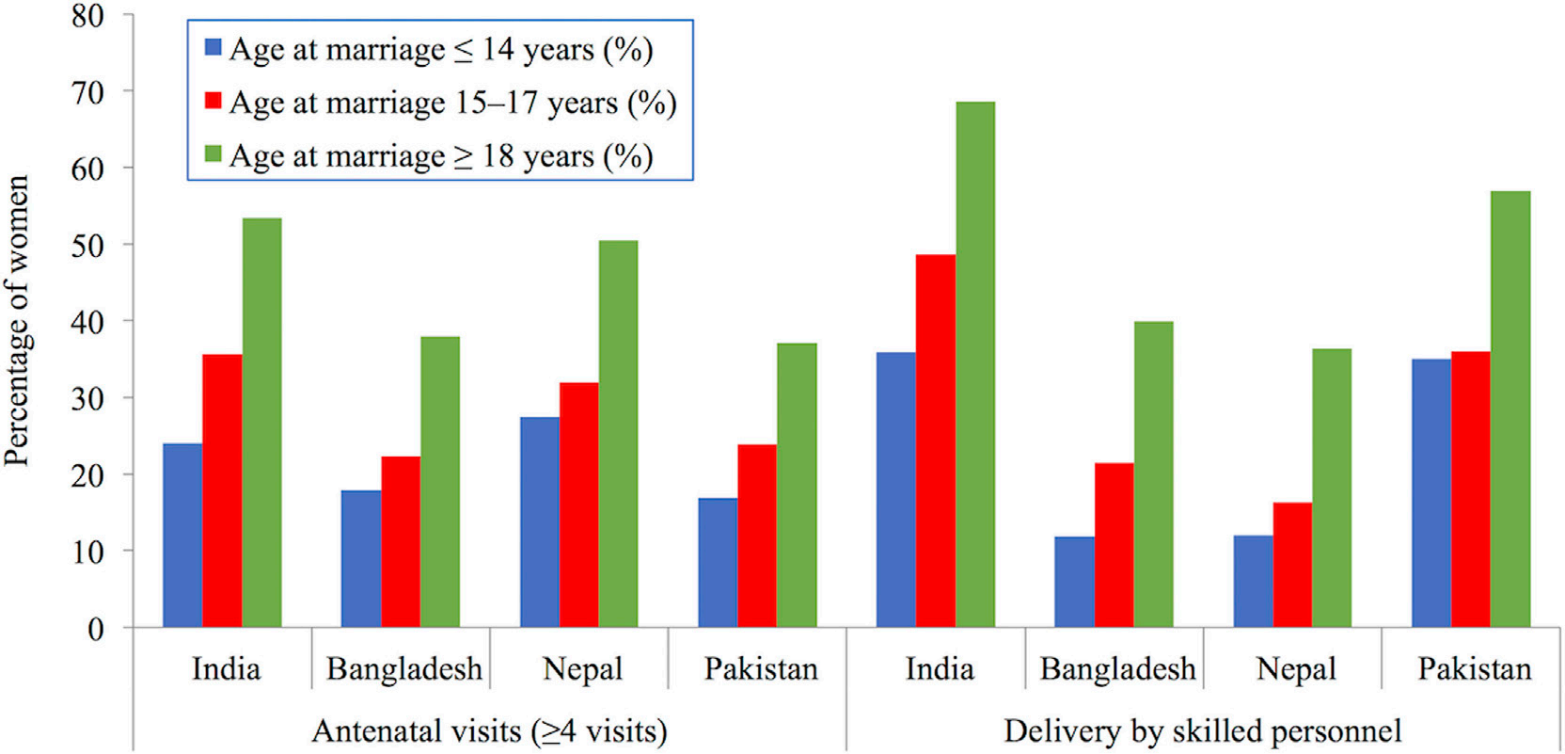
Percentage of women aged 20–24 years accessing adequate ante-natal and delivery services, by age of marriage in South Asia. Demographic Health Surveys data used from following surveys: India (2005–2006), Bangladesh (2007), Nepal (2006), and Pakistan (2006–2007). Data taken from Ref. [(39), (Table 2)].

Much of the public health research focuses on an early age at childbearing, the adverse health outcomes from which are partly attributed to young married women having lower access to contraception, ante-natal care, and delivery by skilled health care workers or in health care facilities (39). In the South Asian context, early childbearing is strongly linked to early marriage. Using DHS data produced by Godha et al., Figure 6 shows that early marriage is strongly associated with a lower likelihood of accessing adequate ante-natal and delivery care (39, 43). The survey data come from the following countries and years: India (2005–2006), Bangladesh (2007), Nepal (2006), and Pakistan (2006–2007).

Early childbearing is associated with high maternal morbidity during pregnancy and labor. An analysis of 312,297 deliveries across 29 countries (including India and Nepal) participating in the WHO Multi-country Survey on Maternal and Newborn Health found that compared with mothers aged 20–24 years, adolescent mothers under 16 years of age had higher risks of cesarean section delivery, eclampsia (seizures which can lead to coma, cerebral hemorrhage, and cardiac arrest), puerperal endometritis (uterine infection), and systemic infections (44). The magnitude of the risk was generally higher for the youngest mothers, aged 15 years or less. Early sexual initiation and childbirth are also associated with a higher risk of developing fistula (involuntary urinary incontinence and/or leakage of feces), a debilitating condition which often leads to social exclusion (45, 46). Comprehensive data on the magnitude of this problem are lacking in South Asia, although one study of >5,000 women in Pakistan estimates vesico-vaginal fistula affects 3.9 per 1,000 women and 4.5 per 1,000 parous women (47).

#### Nutritional Status

Younger mothers are also more likely to be undernourished. In India, a third of adolescent girls marrying and giving birth <18 years were categorized as “thin” [body mass index (BMI) of <18.5] and 58% had severe to mild anemia. Overall, girls married under-age were twice as likely to be undernourished as those married at age 25 years or above (48). This high rate of malnutrition among young mothers is a critical public health concern because adolescence is a period of nutritional vulnerability due to rapid growth and development. In healthy individuals, an estimated 50% of adult weight and more than 15% of adult height is gained between the years of 10–19 years (49). By beginning their reproductive careers during this critical period of physical growth, before biological maturity, undernourished adolescents are likely to attain a shorter adult stature than expected, and hence an increased risk of health complications (50, 51). In Bangladesh, a study comparing 700 pregnant and non-pregnant adolescents found that pregnancy and lactation curtailed linear growth and resulted in weight loss and depletion of fat and lean body mass of young girls (52). Pregnancy and lactation are also likely to increase the nutritional vulnerability of adolescent girls by depleting fat stores and micronutrients (52).

#### Mortality

Many of these pregnancy- and childbirth-related morbidities carry a risk of death. Many older studies have found a higher maternal mortality ratio (defined as deaths to mothers during pregnancy, childbirth or in the 42 days following delivery from pregnancy or childbirth-related causes, per 1,000 births) in mothers under the age of 20 years (53). However, recent studies show that this is not as high as previously thought. There is a relatively small excess adolescent risk, with the lowest risk among 20–24 year old mothers, and then sharply increased risks corresponding to greater maternal age (54, 55). Among adolescents, the risks are higher for younger women, particularly those under age 16 years (44, 56). Young mothers are particularly vulnerable to pregnancy-related morbidity such as death from eclampsia (57).

There are a number of reasons why young wives and mothers might be at higher risk of maternal morbidity and mortality: physiological factors, bio-demographic factors, and socio-environmental factors. Physiological factors include biological immaturity in women which could account for conditions such as cephalo-pelvic disproportion. Bio-demographic factors include parity (how many pregnancies the mother has previously had) which is important because young mothers are more likely to be nulliparous (having their first baby). First pregnancies are also at higher risk than second and third, particularly from eclampsia. Socio-environmental or behavioral factors include wealth, education, access to ante-natal care, contraception, health facilities, and so on.

It is difficult to disentangle these influences, but the fact that in some (but not all) analyses the excess mortality for adolescent wives and mothers disappears when bio-demographic and socio-environmental factors are controlled suggests that the main drivers of excess mortality among young mothers may fall into these categories (44, 56, 58). The fact that adolescent mothers are less likely to be educated, wealthy, urban dwellers means that they are less likely to access the ante-natal care which can help them negotiate a safe path through pregnancy and childbirth.

### Child Outcomes

#### Health Outcomes

The health consequences of maternal under-age marriage also extend to their children. Poor maternal nutritional status is in turn associated with a poor start in life for children who are more likely to experience other social and health penalties (59, 60). An analysis of over 19,000 mother–child dyads from the Consortium for Health Orientated Research in Transitioning Societies study in Brazil, Guatemala, India, the Philippines, and South Africa found that in comparison with mothers aged 20–24 years, younger maternal age at first birth (≤19 years) had a 20–30% increased risk of low-birth-weight (LBW) and pre-term birth, a 30–40% increased risk of stunting (low height-for-age) of children at 2 years, and failure of children to complete secondary schooling (61). The risk of offspring morbidity also increases since younger mothers produce lower volumes of breast-milk and colostrum, which contains antibodies critical for building infant immunity (62, 63).

#### Nutritional Status

In India, at first glance, studies find an inconsistent association of maternal marriage age with childhood stunting and underweight. One study found children born to women who married under-age were 20% more likely to be stunted and underweight than those born to older mothers, even after controlling for demographic characteristics and maternal nutritional status (61). In contrast, another study showed maternal marriage age was only weakly associated with children’s stunting and underweight (low weight-for-age) (64). However, this study also identified five strong predictors of childhood under-nutrition, which are in themselves associated with maternal under-age marriage. These include short maternal stature, lack of maternal education, low household wealth, poor dietary diversity, and maternal underweight. Hence, children of mothers who experience early childbirth are likely to be at a higher risk of under-nutrition in early life, which is also associated with poorer brain, cognitive and emotional development, and capabilities (65). These factors have enduring physical and mental health and human capital consequences in adulthood.

#### Mortality

**Figure 7:**
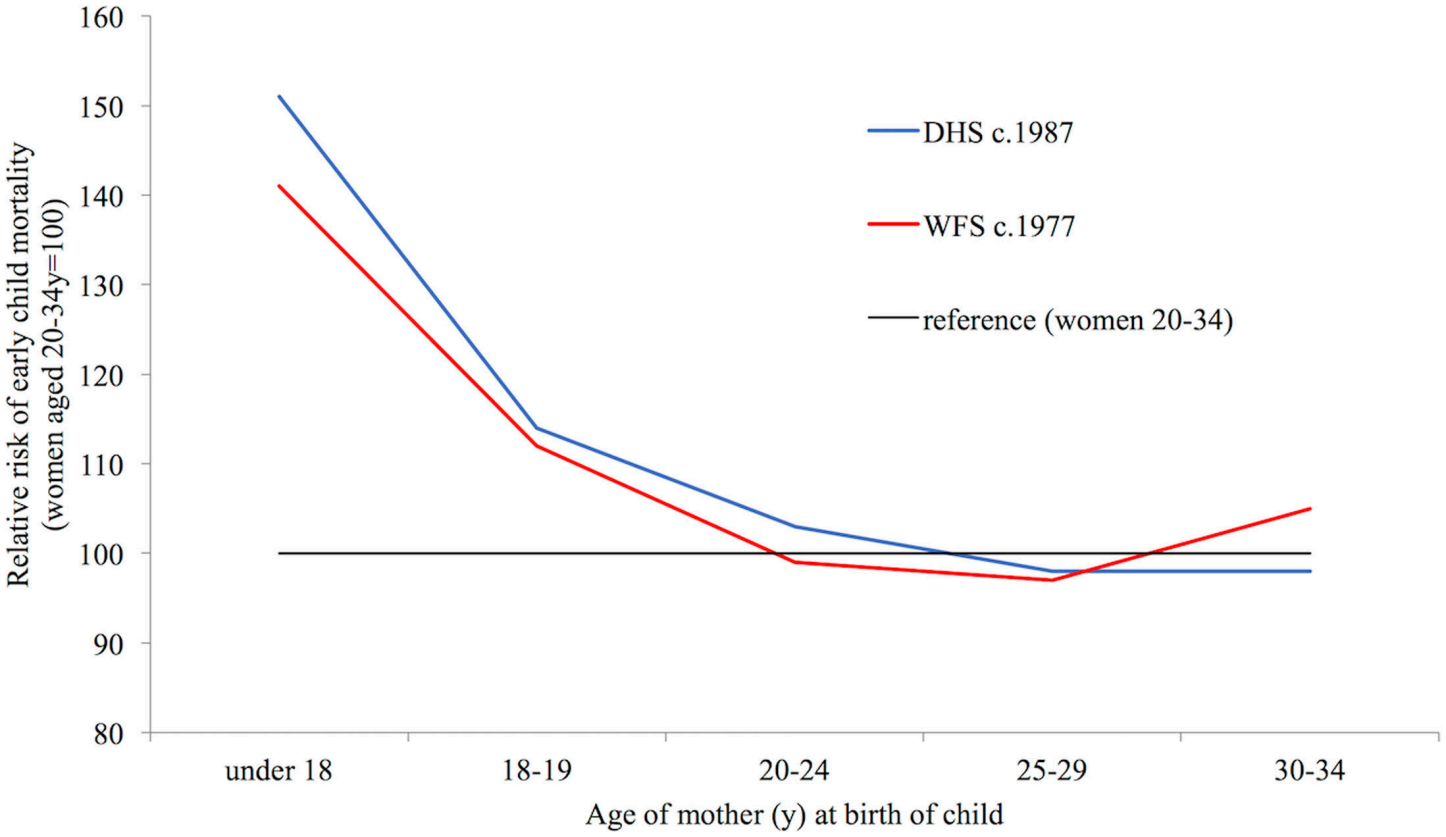
Relative risk of a child dying before age 5, according to mother’s age at its birth. Data taken from Ref. [(66), (Table 2)].

These vulnerabilities also result in higher risks of mortality among the children of younger mothers. According to WFS and DHS data for 18 countries from 1997 to 1987 produced by Hobcraft, compared with the children of mothers aged 20–34 years, children who were born when their mothers were under 18 years of age were 50% more likely to have died before the age of 5 years (Figure 7) (66). Although these are not recent data, the fact that the child mortality rate among women aged 20–34 years declined from 127 to 89 between the two survey years with no reduction in the age pattern suggests that the pattern is unlikely to have changed much despite further declines in child mortality (66).

As with maternal outcomes, higher risks among the children of younger women can depend on bio-demographic, socio-environmental, and physiological factors. As well as being more risky for the mother, first births are also more dangerous for the child. Some analyses have attributed most of the association between young maternal age and infant mortality to the high proportion of first births and shorter birth intervals which often accompany young motherhood (67). The social disadvantage of young mothers may also contribute to higher risks of death among their children (67). Higher infant mortality among young mothers may be also mediated by physiological factors such as feto-maternal competition for nutrients which can lead to pre-term and LBW infants, who have an elevated risk of infant death (44, 58). Studies which look separately at first births demonstrate a clear mortality penalty for young mothers, showing that the disadvantage cannot be completely attributed to first births and short birth intervals. For example, Hobcraft showed that the risk of death before the age of 5 years for children born to women under the age of 20 years in 18 low- and middle-income countries was around 50% higher than to those born to older women among first births, and also among both well-spaced births and poorly spaced births (66). Finlay et al. find that a mortality penalty remains for the first born children of mothers under 18 years of age in comparison with the first born children of older mothers in 55 low and middle-income countries even when controlling for socio-economic status (68).

Similarly, Raj et al. observe a continued infant penalty for motherhood before 18 years in India, Pakistan, and Nepal even when parity, birth intervals, and socio-economic status were controlled (69). This study estimated that motherhood before age 18 years contributed to 11, 12, and 16% of infant mortality in these countries, respectively. Several studies find that the higher risk of dying around birth or in the month after birth, for the children of young mothers compared with those of older mothers, is almost entirely accounted for by the biological mediators of LBW and pre-term birth, but that the continued higher risks of dying later remain even when available biological, demographic, and socio-economic factors are controlled (70, 71). This suggests that the child-care practices of young mothers might be affected through routes difficult to capture in the sort of surveys frequently used; routes which might include female autonomy and decision-making.

The findings reported here have related to the age of the mother at the time of the child’s birth. Although related to age at marriage, it is not always the same thing as women who married young will also have had children at older ages. Few studies examine the effect of early marriage on infant and child mortality while also controlling for age at birth. One study which did control for these factors finds that age at birth and other socio-economic factors partly explain the effect of early marriage on mortality before the age of 5 years and on LBW (72). Therefore, effects of age at marriage on infant mortality could operate partly through age at birth (*via* physiological effects on LBW and prematurity) and partly through socio-economic and female empowerment routes. Below, we discuss these social factors in detail and how they shape different pathways to women’s marriage age. Both are crucial for public health.

### Social Consequences

In contrast to the emphasis of public health and demographic research on age at childbearing, social scientific research focuses principally on the social significance of women’s age at marriage. The following section reviews key themes arising from this literature. Generally, studies find that women who marry earlier are less likely to have opportunities to develop a general sense of overall well-being. This is in part related to lower participation in education, fewer opportunities for employment and training, development of social networks, and broader civic engagement. Together, these outcomes contribute to women’s low status in households and broader society. The key implication for public health is that these outcomes are likely to be associated with poor knowledge of the factors increasing maternal and child poor health, under-nutrition, and mortality. Women who marry at an earlier age are also more likely to have less knowledge about and lower access to contraception, and hence weak control over their fertility and less health care from a trained provider.

#### Well-being

According to UN statements, under-age marriage constrains overall well-being by denying girls their childhood (1, 3). However, marriage not only accelerates the transition to “womanhood,” it also reduces opportunities for personal, emotional, and psychosocial development during the critical middle phase of adolescence (73). During adolescence one’s identity, selfhood, and sense of place in society are developed, often in relation to the broader culture and customs (74). Critical knowledge about reproductive and sexual health is also gained during these years, either through school or peer groups (75). Lack of this knowledge, and the implications this psychosocial development has for autonomy, empowerment, and agency, is likely to be associated with adverse health outcomes, for both young mothers and their children.

In patriarchal societies, such as in South Asia, these transitions are not strictly defined by age. They reflect the social roles expected of girls, and also the timing of sexual and physiological development. These factors may also function as a “social signal” for the readiness for marriage (76). For girls, social roles are likely to be restricted to the domestic sphere, to being a daughter, wife, home-maker, and mother (42). For young mothers, the fulfillment of these diverse domestic roles often implies physical and social isolation from the maternal household, peers, and wider society, which may have knock-on effects on their mental health (e.g., susceptibility to depression), nutritional status, and their own and their children’s health outcomes (42).

#### Education

**Figure 8:**
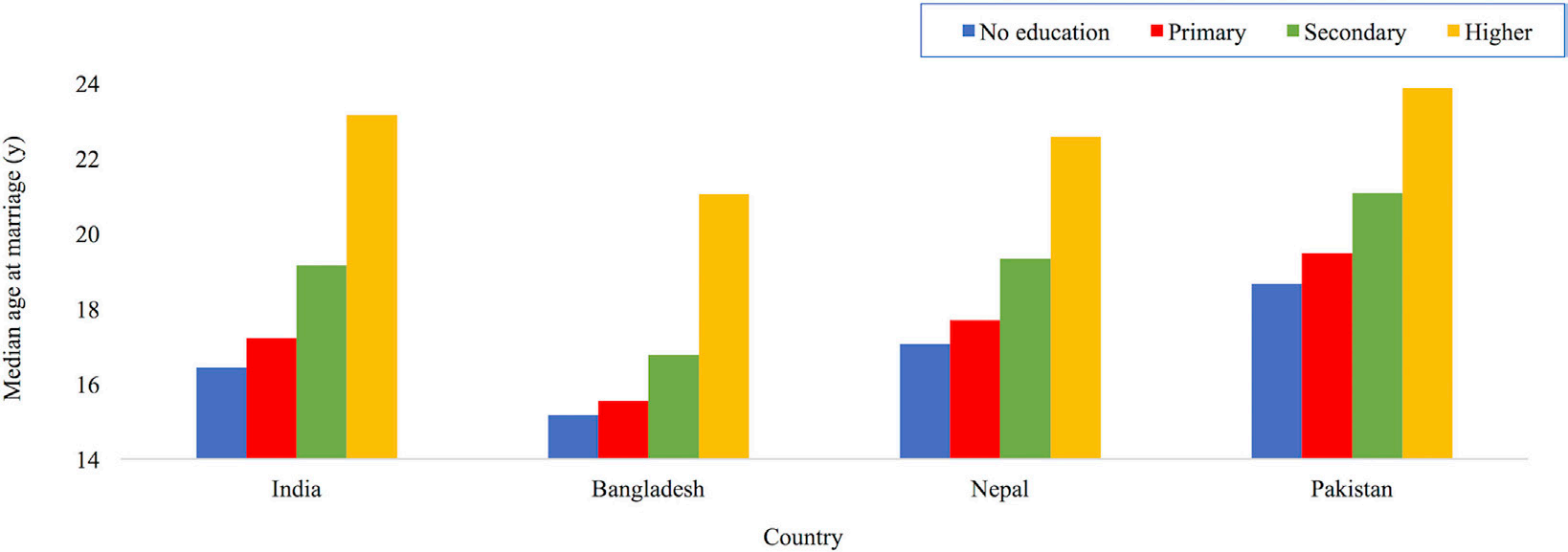
Median age at first marriage by level of education of women aged 25–49 years in South Asia. Drawn with permission using data from Ref. [(16), (Table 7)].

Education plays a crucial role in women’s lifecycle by shaping the timing of key events. In South Asian societies where there is usually a “choice” between education and other life opportunities, getting married generally means leaving school (1). Estimates using the Matlab Health and Socio-economic Survey of >2,000 women aged 25–44 years in Bangladesh confirm this, showing that each additional year of delay in the age of marriage would increase schooling by 0.22 year (77). Figure 8 uses DHS data produced by MacQuarrie from the most recent surveys: India (2005–2006), Bangladesh (2014), Nepal (2011), and Pakistan (2012–2013). It shows that for women aged 25–49 years, the median age at marriage increases with the level of education completed across South Asia (16). Since childbearing usually follows marriage in these societies, it too is inversely related to education level (16).

**Figure 9:**
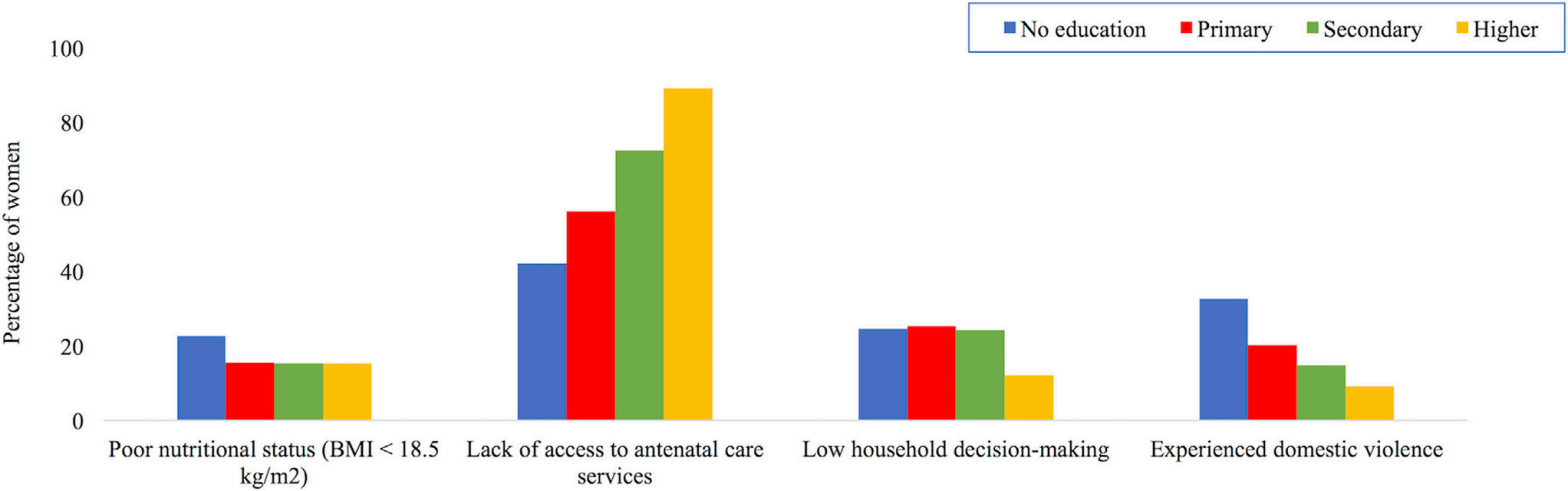
Maternal outcomes by education level for women aged 15–49 years in Nepal, 2011. Data taken from Ref. (15).

Studies consistently find that women with lower levels of education are also more likely to experience multiple vulnerabilities. Figure 9 uses data on women aged 15–49 years from the recent 2011 DHS report from Nepal (15). It shows that in comparison with women with greater levels of education, women with lower educational attainment are more likely to have poor nutritional status (BMI < 18 kg/m^2^) and lower access to ante-natal services. These less educated women are also less likely to participate in household decision-making (regarding own health care, purchases, and visiting relatives) and to have experienced violence. There are similar implications of lower maternal education for children’s malnutrition and survival. For example, in Nepal the mortality rate for children under 5 years of age born to women with no education was more than double that of children born to mothers with secondary or higher education: 73 deaths per 1,000 live births compared with 32 deaths per 1,000 live births (15). Similar associations are apparent in Bangladesh, India, and Pakistan (14, 20, 78).

There are also trans-generational penalties of less education. In the context of public health, education is best understood as a key component of maternal phenotype or “capital,” the physiological niche to which each child is exposed to during the start of life (79, 80). Hence, if less education is a “consequence” of maternal under-age marriage, then this cycle of disadvantage may be perpetuated through lower schooling and under-age marriage of daughters, who are likely to experience similar health consequences as their mothers, and pass them onto the next generation (81). Studies suggest there may be a “threshold” effect of education, although the minimum level of schooling required to achieve improved trans-generational outcomes differs across countries (69, 82). For example, Bates et al. find that in Bangladesh, 5 or more years of maternal education was associated with substantial delays in daughters’ age at marriage (81). Studies have found similar associations between maternal education and daughter’s marriage age in Nepal and Pakistan (83, 84).

Education, and by association the level of schooling completed, are crucial for public health because they are perceived to provide the knowledge, confidence, and agency required to make informed decisions related to maternal and child health, nutritional status, and survival (85). Greater levels of maternal education and literacy are expected to improve the ability of mothers to access and demand appropriate services in support of better growth and development of their children (86, 87). For example, mothers who participated in women’s groups across South Asia not only improved their literacy, but were also able to use information on maternal and child nutrition and healthcare to decrease the proportion of children born with LBW (88, 89). Education is also considered to facilitate greater autonomy to negotiate new and less gendered roles in society, age at marriage, and childbearing (13). Maternal ability to adopt behaviors that children need, including early stimulation, is also expected to be enhanced with greater years of schooling (90). Presumably, greater paternal educational attainment may lead to similar positive outcomes. However, data to compare the independent associations of parental education with daughter’s age at marriage are not routinely collected in studies.

#### Empowerment and Autonomy

Together, lack of education and under-age marriage contribute to lower empowerment for women at the individual level throughout the life-course (91). Husbands and mothers-in-law may exert greater control over younger women, who, being less educated, may be less able to assert themselves (92). Das Gupta terms this subordinated position of young mothers, especially in joint families, as “double powerlessness” (93). Being female and of a younger age limits their ability to exercise autonomy during their childbearing years. For example, young mothers’ lack of control over their own fertility increases the risk of numerous negative maternal reproductive health and child-survival outcomes (94).

Domestic violence is another aspect of low empowerment related in part to young women’s lower ability to resist and refute. Compared with women who married after 18 years, those married under-age are more likely to experience physical or sexual violence (95). For example, interviews with 8,314 young women aged 20–24 years across five Indian states with the highest prevalence of under-age marriage^4^ found women married after 18 years of age were 1.24 times less likely than women married under-age to accept physical violence, and approximately 0.6 times less likely to have experienced marital physical or sexual violence (96). Another analysis of DHS data on women aged 25–49 years from Bangladesh, India, Nepal, and Pakistan over the past two decades also finds that women who married at a younger age were more likely to experience violence than those who married at an older age (16).

There is likely to be a two-way association between early marriage and poor mental health, and together, these factors have knock-on effects on a range of adverse maternal and child outcomes (97). Broadly, research finds that adolescence is a crucial developmental stage, with 50% of mental disorders presenting by the age of 14 years (98). Girls who marry during adolescence are also more likely to be experiencing the physical and emotional effects of pubertal change, which have their own implications for mental health (99). Early marriage and childbearing, along with gynecological morbidity related in part to pregnancy-related factors are likely to further stress mental well-being (100). This overall “gendered disadvantage” in social roles and status experienced by women has been associated with common mental issues such as depression, stress, and other neurotic disorders (97, 101). For example, a cross-sectional survey from 2001 to 2003 of 3,000 women aged 18–45 years in Goa, India found indicators of gender disadvantage such as an early age at marriage and childbearing, low levels of decision-making autonomy, family support, and sexual violence by husbands increased the prevalence of mixed-anxiety depressive disorder (102). Another qualitative sub-study of the rural Pune Maternal Nutrition Study conducted 12 separate focus group discussions with young mothers and fathers, grandmothers and grandfathers in 1998 (103). It found that young mothers had poor nutritional status and experienced anxiety and depression because their minimal influence over the allocation of resources restricted access to health services, and social isolation prevented them from caring for their children’s health and education. Studies also find that violence experienced in marital homes is related to mental ill-health, including women practicing self-immolation (104).

#### Low Social Status

Age at marriage is likely to shape women’s empowerment and agency within households and their status in the broader community. Studies find that the younger a women marries, the more likely she is to have lower status in each of these hierarchies (105). Smith et al. (106) estimated that if women had equal social status to men in households and communities, the prevalence of underweight children under 3 years in South Asia would decrease by 13.4 million (13%).

**Figure 10:**
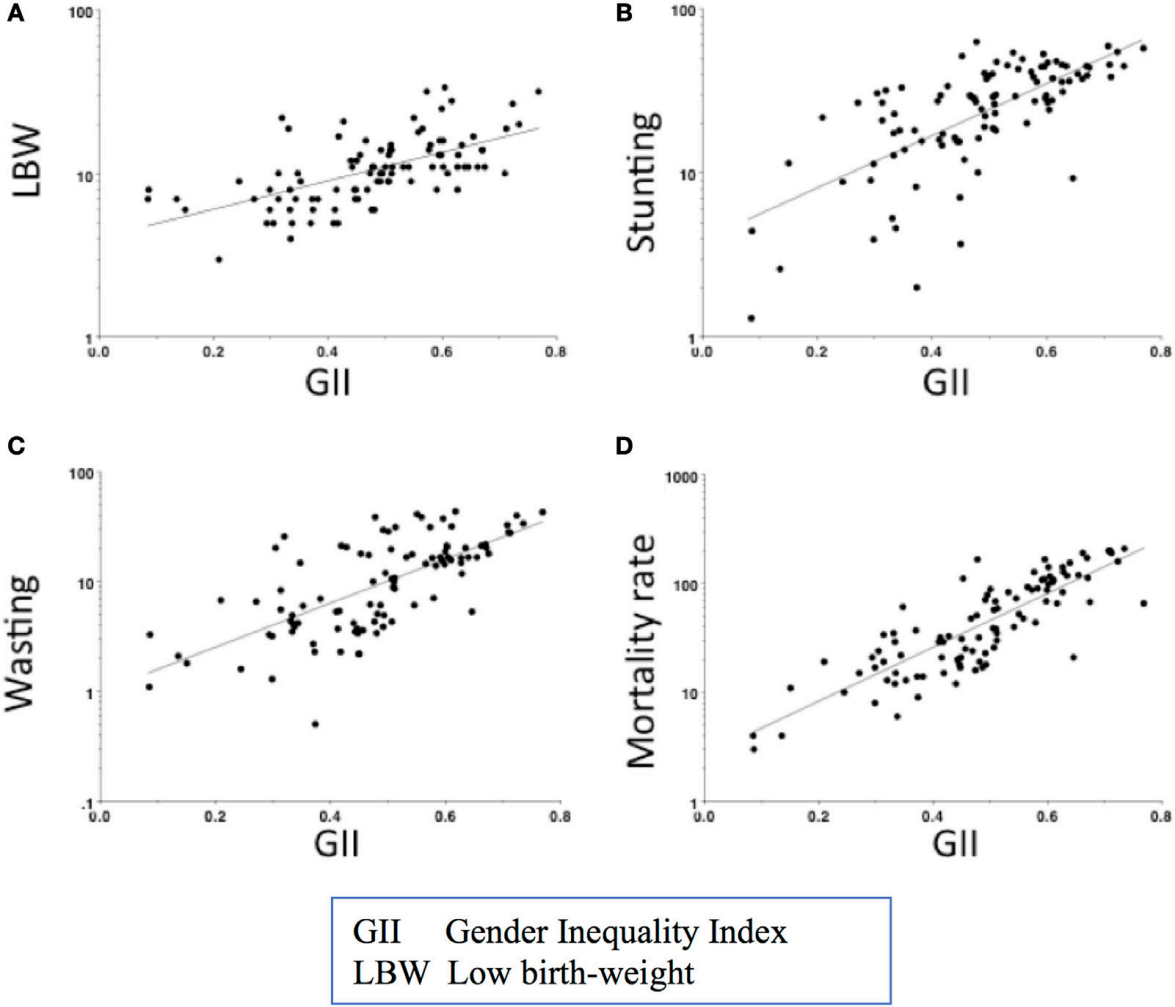
Associations of the gender inequality index with **(A)** low-birth-weight, child **(B)** stunting, **(C)** wasting, and **(D)** mortality across 96 countries. Graph reproduced from Ref. [(109), Figure 1].

At the broader level of society, gender norms and practices also shape the social institutions that structure daily life, including health care and education. Studies have found that women’s lower social status relative to men, as measured by the gender inequality index (GII)^5^, has adverse associations with infant and child mortality and malnutrition (108, 109). The four South Asian countries included in this review rank low on the GII. In 2015, out of 188 countries, they ranked 119 (Bangladesh), 125 (India), 115 (Nepal), and 130 (Pakistan) (107). They also had high rates of child mortality and malnutrition (110, 111). Figures 10A–D show data produced by Marphatia et al. in 2016 on the associations of GII with LBW, child malnutrition, and mortality across 96 countries (109). This study found societal gender inequality explained 36% of the variance in LBW. The GII was also more predictive of LBW than national wealth, measured by per capita Gross Domestic Product (GDP). Independent of GDP, GII also explained 10% of the variance in wasting (low weight-for-height) and stunting, and 41% of the variance in child mortality.

Simulations suggest that reducing societal gender inequality would benefit child outcomes most strongly in the poorest countries. Shifting from the 90th to 50th GII centile in a poor country (10th centile of GDP) would decrease the prevalence of LBW by 4%, stunting by 10%, and childhood mortality by 54% (109). To achieve similar gains through economic growth alone, these low-income countries would effectively need to become middle-income, shifting to the 50th centile of GDP.

The social consequences of under-age marriage are likely to accumulate over time, reducing the very maternal phenotypic components that are key to maternal and child development and health outcomes. Next, we review whether certain factors predispose girls to marrying early. We consider why the practice of under-age marriage continues despite growing evidence of its trans-generational consequences.

## Predictors of Under-Age Marriage

In the previous section, we have noted that some of the negative outcomes for under-age brides and their children might operate through relative socio-cultural disadvantage. In the following section, we discuss the ways in which these factors can lead to under-age marriage in the first place.

### Socio-Cultural Factors

Each of the four South Asian countries included in this review has complex cultural dynamics that might underlie overt preferences for women’s under-age marriage. We highlight key themes from diverse literature on women’s marriage age rather than providing a detailed account of each country’s social context. However, we do use country-specific examples to illustrate key points.

Studies across different disciplines refer to the “socio-cultural norms, customs, and beliefs” shaping decisions relating to marriage age. Studies often use a “cost-benefit” framework to explain the “trade-offs” or penalties for marrying daughters at a particular age. However, Bicchieri et al. point out that many studies do not clearly define the term “social norms,” suggesting instead that “moral rules” better describe how behavior relating to marriage age is governed in societies (112). These codes of conduct and beliefs over credible life options lead people to conform to normative social preferences relating to the age at which girls should marry.

Here, the anthropological literature is helpful in further explaining the significance of the normative beliefs underpinning the practice of early marriage. Kneller defines “culture” as custom, and “societies” as people practicing the customs (74). Both of these aspects play critical roles in the forming of personality because culture is largely internalized and modified by individuals depending on the agency available to them (74). In his seminal anthropological study, Marcel Mauss argues that the person cannot be detached from their broader social structures, hierarchies (socio-economic and gender), and caste/class systems (113). The point, as Vaitla et al. also argue in their 2017 review paper, is that norms and behaviors relating to expected (unequal) social roles and status are deeply rooted in local culture, which in part also shapes individual identity (114). For example, a study conducted in 1990 of 13,200 daughter–mother dyads across 14^6^ Indian states found the sense of “self” was is in part shaped through interactions with various familial, socio-economic, and ecological factors (115). This collective formation of individuals may serve to maintain, rather than challenge, prescribed gender norms.

Within this context of South Asia’s collective societies, people, and their actions are perceived to be socially embedded. Here, studies suggest that as long as families (as opposed to the welfare state) are the main providers of social protection for women, social norms are likely to continue to influence the age at which women marry (116, 117). Broadly speaking, the role and primary identity of a woman in such social contexts are defined by her purpose in life as a “wife, daughter-in-law, and mother.” Hence, the principal “option” in life for women may be marriage. Social norms will thus continue to shape the age at which this is likely to occur and will also influence other opportunities in life such as education.

Historical records suggest that girls’ under-age marriage is not a new phenomenon in contemporary South Asian society. For example, in India, the practice of child marriage, or *Kanya Dan* (gift of a daughter, in Sanskrit), and the social importance and familial pride and prestige attributed to it, is believed to originate in Hindu religious texts (Dharmasutras and Smiritis) in 600 AD. These scriptures warned of the social and religious consequences for parents who failed to marry their daughters soon after menarche [Kapadia DM 1966 cited in Ref. (19)]. The custom of under-age marriage may also originate from socio-cultural practices with patrilineal households desiring to assimilate women from other families into their households [Karve I 1965 cited in Ref. (19)]. An early age at marriage may ensure a bride’s loyalty to her husband’s family. In turn, she would be bound by these very ties. Her low level of education, autonomy, and empowerment may also contribute to shaping her behavior in her marital home. A broader spousal age-gap would also facilitate this “character molding” of younger brides, who are likely to be more responsive to these practices (92).

Whether the practice of under-age marriage in South Asian societies overtly relates to these historical religious dictats is not always clear. However, there is evidence that marrying “early” may be perceived to have benefits whereas marrying “too late” may have social consequences for not only girls but also their families. Several studies provide support for what Maertens terms as the social “institution of early marriage” (p. 1) (118). Caldwell et al.’s 1980 study of 5,000 women in rural Karnataka, India found that although there was a slow shift away from very young child marriages, parents still married their daughters before 18 years because socially this cleared the way to find a bride for their son (17). Maertens, in her 2007–2008 study of over 1,800 individuals in three villages across Maharashtra and Andhra Pradesh states in India finds that failing to adhere to the perceived “ideal” age of marriage (<18 years) in the wider community may lead to criticism and social exclusion which eventually impacts the marriageability of other children, especially girls, in the household (118).

Studies also find that menarche and signs of physical development precipitate under-age marriage because of parental perception of “readiness for marriage” (9, 76). This perception may be related to the expected virginity of prospective brides, which continues to be considered a hallmark of respectability across religions. It is the definition of a “good woman” and hence a necessity for marriage. Virginity may thus be perceived to be in greater “jeopardy” after menarche when the onset of sexual maturity is considered to incite unwanted male attention, risking promiscuity, and sexual violence (76, 119). Under these circumstances, families may face losing their “honor” and girls may be considered unmarriageable, thereby imposing further, long-term burdens on households to provide care for their daughters. As explained in the previous section, the key concern for public health is that girls who marry young may not be physiologically ready for early pregnancy and childbirth.

Collectively, these studies suggest that the agency that is used to support the practice of under-age marriage can be understood as the “socially significant quality of action” (120). Delaying women’s marriage age will invariably require changing the norms underpinning the practice of early marriage and also the low status accorded to women in society. Here, Del Franco’s study in rural Southwest Bangladesh finds that tertiary education accords girls the self-confidence to negotiate a delay in the age at which they marry (121). She argues that we need “…a more nuanced meaning of social embeddedness…to acknowledge that girls are not just passive enactors of other people’s interests and desires” (p. 161) (121). While this suggests a shift in social norms, there may be a selection bias in the sample; the girls who attend university are more likely to come from families who are more supportive of education and delaying women’s marriage age.

These aspects of agency and socio-cultural mores are difficult to measure and compare across women, communities, or countries. Nevertheless, in quantitative studies, culture is reflected in several variables. These include religion, ethnicity, caste, and socio-economic status, all of which may shape social beliefs and behaviors around education, marriage age, fertility, autonomy, etc. (115). Table 1 shows that religion partly shape national law relating to minimum marriage age. However, inferring that any one faith is related to the persistent practice of under-age marriage is difficult because girls appear to be married under-age in all religions. A review of 111 countries found that the prevalence of under-age marriage varied greatly, with no discernable pattern by the countries’ predominant religions (122). This is not to say religion is not a predictor of under-age marriage, but rather that it is often tightly interwoven with broader gendered socio-cultural norms, attitudes, and practices.

### Economic Factors

**Figure 11:**
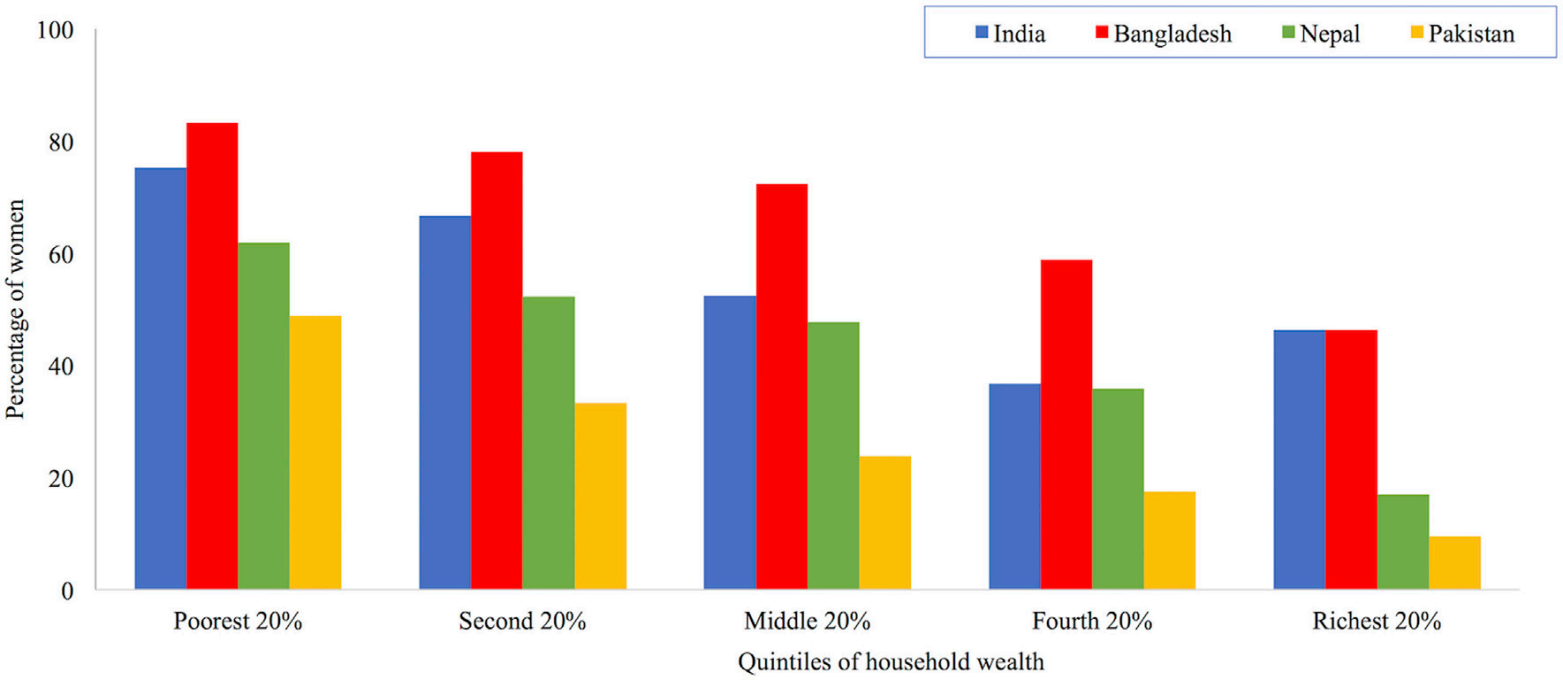
Percentage of women aged 20–24 years married under-age by household wealth in South Asia. Data taken from Ref. (9).

The economic wealth of families, which is often related to socio-cultural status, is a common factor cited in the literature on the predictors of under-age marriage. A recent review of 54 DHS surveys found that girls living in poor households were twice as likely to marry before the age of 18 years when compared with girls in wealthier households (123). MacQuarrie’s analysis of recent DHS data on women aged 25–49 years from Bangladesh, India, Nepal, and Pakistan also finds that women’s age at marriage increases in line with household wealth (16). The United Nation’s Population Fund’s 2012 analysis found a similar trend in the contemporary younger cohort of women aged 20–24 years. The proportion of women marrying under-age decreased as household wealth increased (9). Figure 11 shows the prevalence of under-age marriage by wealth quintiles in South Asia (9). The poorest quintile describes the percentage of women aged 20–24 years from the poorest 20% of households, who were married or in union before their 18th birthday.

Why are households of low-economic status more likely to marry girls at an earlier age? Studies suggest that in resource-constrained households, girls may represent a liability for the limited economic budget and food security for the entire household; the sooner these responsibilities are passed onto the husband’s family the better (19). Douglas (124) explains that in patrilineal societies where family lineage and livelihood depends on sons, more resources are amassed for their development and educational/social advancement. To achieve this, less money is spent on daughters’ education, healthcare, and eventual marriage and dowry (118). However, in South Asian societies, the wealth-marriage age association is complex. Even girls from middle-income families may be “moved on to” their marital homes as early as possible. This is because dowry, which is paid by the woman’s family, increases with the prospective bride’s age, and education level (125). Since the practice of dowry has been illegal since 1961, official data on payment amounts are generally not collected. However, the 2011–2012 Indian Human Development Survey of 42,000 households estimated that the average Indian family gave 30,000 Rupees (approximately $491) in cash for dowry; about 40% also gave televisions and cars (126).

### Rural Residence

**Figure 12:**
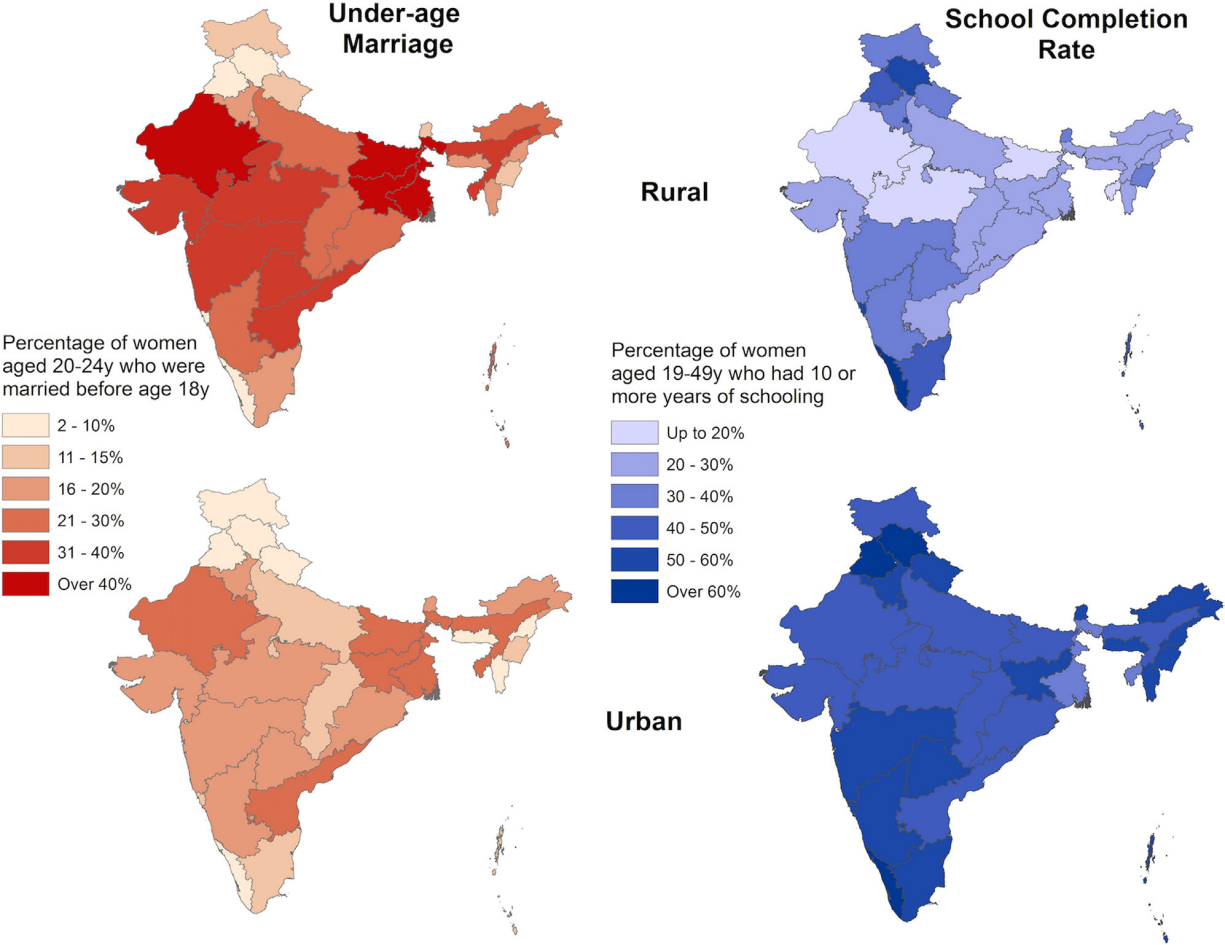
Prevalence of under-age marriage and school completion rates by rural and urban residence, India 2016. Data taken from Ref. (128).

There is geographic heterogeneity in the prevalence of under-age marriage across and within countries. Compared with women in urban areas, those residing in rural areas are generally more likely to come from poorer households, to be married under-age, and to have lower educational attainment. An analysis of 36 Sub-Saharan and South West Asian countries found that women from rural communities who had married early had the greatest deficits in schooling (127). Figure 12 uses data from the recent 2016 National Family Health Survey in India to illustrate that the prevalence of under-age marriage in India is higher in rural than urban areas, especially where the rate of girls schooling has historically been the lowest (128). A similar pattern is found in Bangladesh, Nepal, and Pakistan. Girls residing in rural areas in these three countries also have a higher risk of marrying under-age than their urban peers (9, 129).

Overall, the different predictors of under-age marriage that have been reviewed thus far appear to have a greater rural concentration. There is usually lower availability and access to schools in rural areas (130). This may hasten under-age marriage. Another factor may be the rural economy, which may offer fewer formal employment opportunities to women. Rural areas also tend to have a greater proportion of informal or home-based industries, where young, often uneducated brides are sought to provide labor for free (75). Family farms, upon which these young women also work, contribute to household food security with few economic costs to the household. Furthermore, in poor rural areas where infant mortality is high, young women with longer reproductive careers are particularly sought after to maximize the number of surviving offspring to satisfy household requirements for labor (75).

These factors are also embedded within the broader socio-geographical hierarchy of rural societies, which studies find are more likely to uphold traditional practices of early marriage (131). Singh et al. (96) use Indian NFHS data (2005) on 40,606 women aged 15–59 years from the eight^7^ states with the highest prevalence of under-age marriage and population growth but lowest levels of social and economic growth to identify correlations between different factors. They found rural residence and education level explained 83% of the variability in marriage age. Other factors mapped onto these two predictors. Poorer, lower caste families who tended to reside in rural areas were less likely to educate their daughters and more likely to marry them under-age.

### Education

It is often assumed that although marriage and education are alternative life outcomes for adolescents, marriage largely shapes education in that girls who marry young are forced to leave school. However, there is likely to be a bi-directional association between marriage age and education. Education may also predict the age at which women marry. The role of girls’ education in shaping the timing of key life events such as age at marriage and childbearing is complex. Paradoxically, studies find both fewer and greater years of schooling are associated with under-age marriage. Below, we discuss why these different pathways shape greater susceptibility to under-age marriage.

Overall, data from 2010 to 2014 show that the net enrollment rate (NER)^8^ for girls in primary school was around 94% for India, Bangladesh, and Nepal and 67% in Pakistan (133). However, the NER for secondary school was much lower, at 62% in India and Nepal, 55% in Bangladesh, and 36% in Pakistan (133).

Studies estimate that across 18 of the 20 countries with the highest prevalence of under-age marriage, girls with no education were up to six times more likely to marry as children than girls with secondary education (75). In India, each additional year of education, from primary school onward, reduced the risk of under-age marriage (17, 69). In Bangladesh, Nepal, and Pakistan, there appears to be a “threshold effect” with secondary education being associated with delaying marriage age during early- but not late-adolescence (69). Bongaarts et al.’s analysis using DHS data from 1996 to 2014 for 43 countries including the South Asia region found that the difference in the mean age of first sexual intercourse, marriage, and childbearing is greater between girls with secondary and primary education than the difference between girls with primary and no education (13). This study also found that the overall increase in the educational attainment of girls was accompanied by slower than expected increases in the age at which girls experienced these key life events (13).

Dropping out of school because of poor performance may also increase the probability of under-age marriage, because households may be less likely to invest limited resources in keeping girls in school (127). Of course, leaving school need not necessarily have to be associated with under-age marriage, unless socio-cultural norms imply a “choice” in life between education and marriage.

Poor performance in school may not necessarily be a marker of academic ineptitude. Studies from Bangladesh, India, and Nepal find the lack of available, accessible, acceptable, and adaptable education of good quality may function as a “push factor” for both low educational attainment and an earlier age at marriage (134, 135). Aikman and Rao find that in school girls are often encouraged to fulfill gendered societal roles of early marriage and childbearing (136). Similarly, Jeffrey and Jeffrey’s (137) study raises important questions about the potential failure of education in transforming gendered social roles,

…How can sending girls to school for a greater number of years empower them if the structures of domination in which they are embedded remain unchanged? Does schooling really expose girls to alternatives permitting them to challenge this hegemony and realm of ideas? (p. 161)

The demands of the contemporary marriage market in South Asia suggest the emergence of a new trend. Higher levels of education are not substantially delaying girls’ marriage age. In this context, education becomes an asset for marriage (138). Research suggests that completing secondary school (age 17 years) enhances the social position of girls and also their families by improving the chances of marrying into a family of higher socio-economic status (hypergamy) (139, 140). A higher level of education is increasingly sought after by the bridegroom’s family because it is perceived to be a sign of greater maturity and capability during the eventual reproductive years (84). Paradoxically, staying longer in school, where gendered roles are emulated by teachers and peers, may perpetuate societal norms of under-age marriage (136, 137).

These contrasting findings suggest we need to better understand what girls can do with increased education in societies where other life opportunities, such as higher education or employment, are not yet widely acceptable or accessible to most women (141). Greater education may also not be improving women’s status at the household or societal level. For example, if the education of girls is principally valued as a social asset for obtaining a future husband, then staying in school longer may not increase agency or change the gendered ideals of marriage (142, 143). Paradoxically, recent evaluations of cash stipend interventions to delay girls’ marriage age through increased education in Bangladesh found they were used by families to pay for marriage-related costs (144, 145). This included higher dowries amounts associated with the higher education levels of girls. These programs did not change the lower value attributed to girls in society and their restricted domestic social roles (145).

### Women’s Low Social Status

As with education, women’s subordinated status in society can be both a predictor and a consequence of under-age marriage (146). Socio-cultural practices are likely to reproduce gender unequal power relations and maintain women’s subordinated status (19). However, notions of gender inequality are complex in South Asian societies. Asymmetries of power lie not only along male–female lines but also among females of different ages, and possibly also varying levels of education (147). Young brides usually have the lowest social status in households (93, 148). Mothers-in-law generally have more authority to put into practice decisions about health care, education, and expenditures, although the primary decision-making tends to remain with men (148). Chodorow explains that it is within this larger context of patriarchy that a microcosm of women being agents in their own subordination takes places (147). In traditional contexts such as rural Bangladesh, where gender inequality persists, Bates et al. (81) find that

…empowerment may sometimes enable women to carry out traditional strategies that reflect their fundamental social and economic insecurity in the family and in society at large [;] strategies that may undermine the health and well-being of women in the next generation. (p. 109)

We still need to better understand why female family members who may have experienced adverse consequences of their own under-age marriage may encourage reproduction of the same pathways for their own daughters and daughters-in-law. The difficulty here for researchers is that women’s status is likely to change over their life-course. It may paradoxically lead to the transition from one of subservience to husband to dominance over her own daughter-in-law. Of course, women of all ages may find the agency to resist these norms, but often only within the limited discursive spaces available to them (149).

Here, systematic reviews of interventions show that laws and communication with community members on the consequences of early marriage are a necessary but insufficient condition for delaying marriage age (150). This may be because social norms related to marriage age are changing very slowly. Delprato et al. (127) find that household decisions to marry daughters under-age reflect socio-cultural norms passed on through generations by social pressure to maintain the *status quo*. Their analysis of DHS data from 36 countries in Sub-Saharan Africa and South West Asia finds that girls are more likely to marry under-age when the community also has high rates of past and current marriages of women below 18 years, high fertility, and low levels of pre-marital sex (an indication of the value the community attaches to girls’ virginity and reflecting safety concerns related to early marriage decisions).

At a societal level, policies which are formal institutionalized arrangements of socio-cultural norms may maintain these practices and women’s subordinated status. The widespread neglect of women’s health and nutrition in national policies not only harm women themselves, but also impose a burden on wider society by contributing to long-term, often trans-generational deficits in health and human capital (151). Since culture is also about how different parts of society are organized, including the structures that uphold them, schools play an important role in enabling the formation of societies along these norms (136). In the widest sense, education includes this very process of forming a person’s mind and character. However, many studies argue that schools often serve to reproduce, rather than challenge, existing gendered norms in societies (152).

In summary, although education and, to some extent, economic opportunities for women are more widely available, countries with deeply entrenched traditional justifications for early marriage are not likely to see an end to this practice without a shift in gendered social norms (153). The inter-relatedness of these factors is complex. Families may be caught between status attainment through idealized gender performance where modesty, segregation, and under-age marriage are praised and modernity, where greater education and later age at marriage are emphasized (154). This makes identifying the key levers of change all the more challenging.

## Discussion

While the importance of under-age marriage is recognized in different academic and policy fields, they also approach the problem from contrasting perspectives. The demographic and public health literatures focus largely on the disadvantages of early childbearing. Conversely, social scientists focus on the human capital consequences of under-age marriage, such as education. These different approaches miss the inherent inter-connection between these issues and the implications for public health of the broader social challenges.

**Figure 13:**
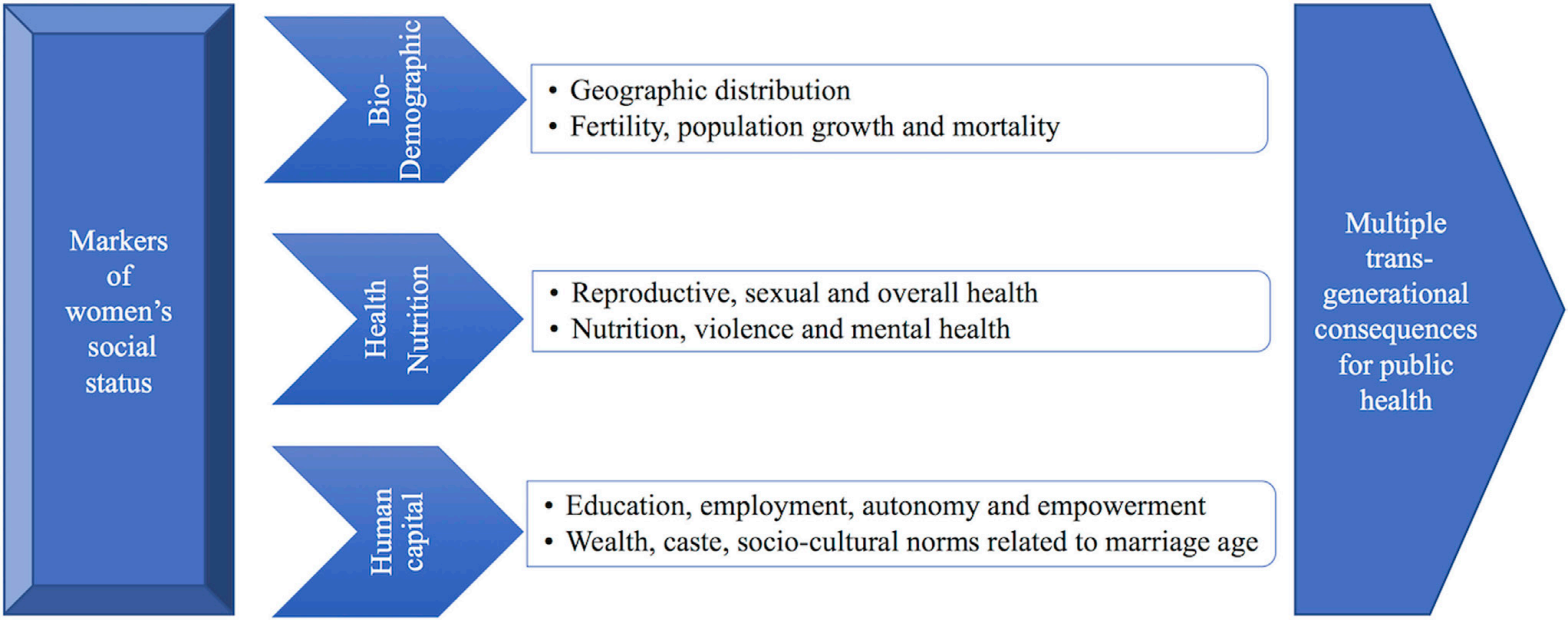
Schematic diagram illustrating the public health implications of women’s under-age marriage. Taken together, these physiological, bio-demographic, and socio-environmental drivers of variable marriage age are markers of women’s low social status. These factors have an adverse effect on women, mothers, and their children. They have a major impact on public health. Adapted from Ref. [(129), Figure 1; (155), Figure 1; (156)].

Our goal in this review has been to draw on these diverse literatures to provide an integrated perspective on variability in women’s marriage age and its implications for public health (Figure 13) (129, 155, 156). Taken together, these factors are markers of women’s low status in society and are likely to have trans-generational consequences. We conclude by discussing some of the implications for research and practice for better understanding the predictors and consequences of under-age marriage in the context of public health.

### Implications for Research

A recent expert group meeting organized by the UN, charity organizations, and academics recognized the need for more research on factors *beyond* gendered norms, education, and poverty contributing to under-age marriage (157). Our review of the literature suggests this would require both a broader scope to research and also different methodological approaches. Research needs to focus on the predictors of *variability* in marriage age to identify the trade-offs of marrying at different ages. This is crucial because factors that contribute to a later marital age may not simply be the inverse of those associated with under-age marriage. Take, for example, the increased participation of girls in education, which has not yet substantially delayed their age at marriage.

Decisions about future life paths, be they education or marriage, are not likely to suddenly appear at one point in time. Such decisions about choices in life may also emerge through cumulative social and biological processes (158). For example, biomedical scientists find a “developmental origins” to adult non-communicable disease and less education (159, 160). Whether factors acting in early life also shape variability in women’s marriage age requires further investigation. Studying these complex associations would require a multi-disciplinary life-course perspective and data on two if not three generations.

A key methodological challenge is disentangling the key drivers of variability in marriage age. Under-age marriage is a marker of several inter-related gender-specific vulnerabilities relevant to public health such as less education, poor nutritional status, and poverty, all of which are concentrated in rural areas (12). There is also a strong correlation between the individual, household, and demographic variables predicting marriage age. The potential bi-directionality and multiple pathways of these associations also renders identification of the predictor and consequence difficult (45). For example, maternal age at childbirth, which is predicted by under-age marriage, is associated with infant mortality; these associations may operate through socio-economic deprivation and biological behavioral factors such as place of delivery, gestation, and birth-weight (59, 71). But socio-economic deprivation and biological behavioral factors may themselves predict under-age marriage.

Another key challenge is comparing and compiling evidence across studies because of their different age categorizations, time frames, and data disaggregation. Furthermore, changes in ecological, economic, and socio-cultural factors during these time periods may not provide an accurate representation of a potential change in the timing of marriage (131). Some factors may also be more important at specific time periods or have a stronger association over time, or the risk related to any type of consequence may accumulate over time. Finally, observational analyses of nationally representative surveys indicate conditional statistical correlations, but they cannot prove causality.

### Implications for Practice

Policies have had some success in decreasing the rate of adolescent fertility (161) and increasing girls’ participation in education (130), both of which are relevant for public health. Estimations suggest the enforcement of even existing contrasting laws on minimum marriage age in South West Asia would increase girls’ schooling by 15% (127). However, increased education, the primary intervention used to delay girls’ marriage age, has had limited success (69). Similarly, systematic evaluations of interventions suggest efforts directly aiming to delay marriage age are more successful than those focusing on related factors such as adolescent sexual and reproductive health, partly because girls have little control over access to these services before and after marriage (162, 163). We do however know that at an individual level, in circumstances where mothers have greater ability to make strategic life choices, and the autonomy, agency, and access to resources required to exercise these choices, children’s nutritional status and health have generally improved (89, 164).

### Conclusion

Our aim was to show that women’s marriage age, and its human capital predictors and consequences, matter for public health. We argue that a broad range of health and social issues, including the low status of women, are likely to be affected by addressing early marriage. Marriage is both a cultural practice, reflecting women’s status in society, and linked to multiple biological, ecological, and geographical factors, each of which is crucial for public health. Marriage is the “gateway” to the multiple health consequences associated with the timing of childbirth. It is also a predictor of human capital penalties, which have their own implications for health. The gaps identified in knowledge and the general ineffectiveness of policies and interventions help explain why the early age at which girls marry is both expected and accepted, and why it changes very slowly (165). Disentangling the broad predictors of marriage age is complex. They are inter-related, and tightly interwoven with socio-cultural norms, broader economic and geographical contexts, and trans-generational developmental processes (123, 166).

## Author Contributions

AM conceived the original idea and developed it with guidance from AR and GA. AM wrote the first draft of the article. AR wrote sections on demography, maternal, and child survival. GA produced the maps and their interpretations. All authors provided detailed feedback on the full manuscript and contributed to revisions.

## Conflict of Interest Statement

The authors declare that the research was conducted in the absence of any commercial or financial relationships that could be construed as a potential conflict of interest.

## Footnotes

^1^Data were not available for all countries and all time-points for the recent cohort of women aged 20–24 because less than 50% of women began living with their spouse for the first time before reaching the beginning of the age-group (14, 15).

^2^The gendered context was measured by the extent to which women participated in household decision-making, accepted/experienced violence, and their spousal age gap (16).

^3^Teenage pregnancies in places where pre-marital intercourse is common are likely to result in higher completed fertility due to a link between fecundity and the likelihood of getting pregnant in the first place, and due to the negative effect of a teenage pregnancy on education and career progression (33). This is not considered to be a major factor in South Asia where pre-marital intercourse remains rare, and it can complicate comparisons of marriage age and fertility between different parts of the world.

^4^Andhra Pradesh, Bihar, Jharkhand, Maharashtra, Rajasthan, and Tamil Nadu.

^5^The Gender Inequality Index is a composite statistic of the loss in potential human development due to disparities between female and male achievements in empowerment (secondary educational attainment and parliamentary representation), economic status (labor market participation), and women’s health (adolescent birth rate and maternal mortality ratio) (107).

^6^Andhra Pradesh, Bihar, Delhi, Gujarat, Karnataka, Kerala, Madhya Pradesh, Maharashtra, Orissa, Punjab, Rajasthan, Tamil Nadu, Uttar Pradesh, and West Bengal.

^7^Bihar, Uttar Pradesh, Madhya Pradesh, Rajasthan, Orissa, Jharkhand, Uttrakhand, and Chattishgarh.

^8^The number of pupils of official school age who are enrolled in education as a percentage of the total children of the official school age population (132).
